# Uncovering Treatment Burden as a Key Concept for Stroke Care: A Systematic Review of Qualitative Research

**DOI:** 10.1371/journal.pmed.1001473

**Published:** 2013-06-25

**Authors:** Katie Gallacher, Deborah Morrison, Bhautesh Jani, Sara Macdonald, Carl R. May, Victor M. Montori, Patricia J. Erwin, G. David Batty, David T. Eton, Peter Langhorne, Frances S. Mair

**Affiliations:** 1 General Practice and Primary Care, Institute of Health and Wellbeing, University of Glasgow, United Kingdom; 2 Faculty of Health Sciences, University of Southampton, United Kingdom; 3 Knowledge and Encounter Research Unit, Mayo Clinic, Rochester, United States of America; 4 Department of Epidemiology and Public Health, University College London, United Kingdom; 5 Centre for Cognitive Ageing and Cognitive Epidemiology, University of Edinburgh, United Kingdom; 6 Institute of Cardiovascular and Medical Sciences, University of Glasgow, United Kingdom; St. Michael's Hospital, Canada

## Abstract

**Background:**

Patients with chronic disease may experience complicated management plans requiring significant personal investment. This has been termed ‘treatment burden’ and has been associated with unfavourable outcomes. The aim of this systematic review is to examine the qualitative literature on treatment burden in stroke from the patient perspective.

**Methods and Findings:**

The search strategy centred on: stroke, treatment burden, patient experience, and qualitative methods. We searched: Scopus, CINAHL, Embase, Medline, and PsycINFO. We tracked references, footnotes, and citations. Restrictions included: English language, date of publication January 2000 until February 2013. Two reviewers independently carried out the following: paper screening, data extraction, and data analysis. Data were analysed using framework synthesis, as informed by Normalization Process Theory. Sixty-nine papers were included. Treatment burden includes: (1) making sense of stroke management and planning care, (2) interacting with others, (3) enacting management strategies, and (4) reflecting on management. Health care is fragmented, with poor communication between patient and health care providers. Patients report inadequate information provision. Inpatient care is unsatisfactory, with a perceived lack of empathy from professionals and a shortage of stimulating activities on the ward. Discharge services are poorly coordinated, and accessing health and social care in the community is difficult. The study has potential limitations because it was restricted to studies published in English only and data from low-income countries were scarce.

**Conclusions:**

Stroke management is extremely demanding for patients, and treatment burden is influenced by micro and macro organisation of health services. Knowledge deficits mean patients are ill equipped to organise their care and develop coping strategies, making adherence less likely. There is a need to transform the approach to care provision so that services are configured to prioritise patient needs rather than those of health care systems.

**Systematic Review Registration:**

International Prospective Register of Systematic Reviews CRD42011001123

*Please see later in the article for the Editors' Summary*

## Abstract 2

In a systematic review of qualitative research, Katie Gallacher and colleagues examine the evidence related to treatment burden after stroke from the patient perspective.

*Please see later in the article for the Editors' Summary*

## Editors' Summary

**Background:**

Every year, 15 million people have a stroke. About 5 million of these people die within a few days, and another 5 million are left disabled. Stroke occurs when the blood supply of the brain is suddenly interrupted by a blood vessel in the brain being blocked by a blood clot (ischemic stroke) or bursting (hemorrhagic stroke). Deprived of the oxygen normally carried to them by the blood, the brain cells near the blockage die. The symptoms of stroke depend on which part of the brain is damaged but include sudden weakness or paralysis along one side of the body, vision loss in one or both eyes, and confusion or trouble speaking or understanding speech. Anyone experiencing these symptoms should seek immediate medical attention because prompt treatment can limit the damage to the brain. In the longer term, post-stroke rehabilitation can help individuals overcome the physical disabilities caused by stroke, and drugs that thin the blood, reduce blood pressure and reduce cholesterol (major risk factors for stroke) alongside behavioral counseling can reduce the risk of a second stroke.

**Why Was This Study Done?:**

Treatment for, and rehabilitation from, stroke is a lengthy process that requires considerable personal investment from the patient. The term “treatment burden” describes the self-care practices that patients with stroke and other chronic diseases must perform to follow the complicated management strategies that have been developed for these conditions. Unfortunately, treatment burden can overwhelm patients. They may be unable to cope with the multiple demands placed on them by health-care providers and systems for their self-care, a situation that leads to poor adherence to therapies and poor outcomes. For example, patients may find it hard to complete all the exercises designed to help them regain full movement of their limbs after a stroke. Treatment burden has been poorly examined in relation to stroke. Here, the researchers identify and describe the treatment burden in stroke by undertaking a systematic review (a study that uses predefined criteria to identify all the literature on a given topic) of qualitative studies on the patient experience of stroke management. Qualitative studies collect non-quantitative data so, for example, a qualitative study on stroke treatment might ask people how the treatment made them feel whereas a quantitative study might compare clinical outcomes between those receiving and not receiving the treatment.

**What Did the Researchers Do and Find?:**

The researchers identified 69 qualitative studies dealing with the experiences of stroke management of adult patients and analyzed the data in these papers using framework synthesis—an approach that divides data into thematic categories. Specifically, the researchers used a coding framework informed by normalization process theory, a sociological theory of the implementation, embedding and integration of tasks and practices; embedding is the process of making tasks and practices a routine part of everyday life and integration refers to sustaining these embedded practices. The researchers identified four main areas of treatment burden for stroke: making sense of stroke management and planning care; interacting with others, including health care professionals, family and other patients with stroke; enacting management strategies (including enduring institutional admissions, managing stroke in the community, reintegrating into society and adjusting to life after stroke); and reflecting on management to make decisions about self-care. Moreover, they identified problems in all these areas, including inadequate provision of information, poor communication with health-care providers, and unsatisfactory inpatient care.

**What Do These Findings Mean?:**

These findings show that stroke management is extremely demanding for patients and is influenced by both the micro and macro organization of health services. At the micro organizational level, fragmented care and poor communication between patients and clinicians and between health-care providers can mean patients are ill equipped to organize their care and develop coping strategies, which makes adherence to management strategies less likely. At the macro organizational level, it can be hard for patients to obtain the practical and financial help they need to manage their stroke in the community. Overall, these findings suggest that care provision for stroke needs to be transformed so that the needs of patients rather than the needs of health-care systems are prioritized. Further work is required, however, to understand how the patient experience of treatment burden is affected by the clinical characteristics of stroke, by disability level, and by other co-existing diseases. By undertaking such work, it should be possible to generate a patient-reported outcome measure of treatment burden that, if used by policy makers and health-care providers, has the potential to improve the quality of stroke care.

**Additional Information:**

Please access these Web sites via the online version of this summary at http://dx.doi.org/10.1371/journal.pmed.1001473.

- The US National Institute of Neurological Disorders and Stroke provides information about all aspects of stroke (in English and Spanish); its Know Stroke site provides educational materials about stroke prevention, treatment, and rehabilitation including personal stories (in English and Spanish); the US National Institutes of Health SeniorHealth website has additional information about stroke
- The Internet Stroke Center provides detailed information about stroke for patients, families, and health professionals (in English and Spanish)
- The UK National Health Service Choices website also provides information about stroke for patients and their families, including personal stories
- MedlinePlus has links to additional resources about stroke (in English and Spanish)
- The UK not-for-profit website Healthtalkonline provides personal stories about stroke
- Wikipedia provides information on the burden of treatment and on the normalization process theory (note: Wikipedia is a free online encyclopedia that anyone can edit; available in several languages)

## Introduction

### The Concept of Treatment Burden

‘Treatment burden’ is a novel concept describing the self-care practices that patients with chronic disease must perform to enact management strategies and respond to the demands of health care providers and systems. Individuals will vary in their capacity to accommodate and enact such practices, which may have a marked impact on patient functioning and well-being [1]–[4], and on adherence to management plans [5]–[8]. Nonadherence to management strategies by patients with chronic disease is a global health problem [9] and has the potential to lead to negative outcomes for patients such as increased morbidity and wasted expenditure for health care systems [1],[10]. There is growing interest in the concept of treatment burden internationally [1]–[4], and it has been hypothesized that treatment burden can overwhelm patients, exceeding their coping threshold and leading to poor adherence to therapies [7],[11],[12]. Coping thresholds are likely to vary substantially between patients, depending on environmental factors such as social support and financial constraints [5],[13],[14], and on internal factors such as health literacy and resilience [15],[16].

There has recently been a growing interest in the development of a patient-reported outcome measure of treatment burden in chronic disease, to be utilised by health care providers alongside consideration of patient capacity [3],[4]. It is well documented that the initial steps of development of such a measure are to explore the patient experience through qualitative methods [17]. This review explores the features of treatment burden in one chronic disease, stroke, but we expect many of the key concepts identified would be generic and relevant to those with other chronic diseases and multimorbidity [18].

### Treatment Burden in Stroke

Cerebrovascular disease (stroke) is the leading cause of long-term disability in both the UK and the US [19],[20]. Global projections to the year 2020 indicate that this disease burden will increase, in both westernised and resource-poor countries [21]. It is well documented that treatment for, and rehabilitation from, stroke can be an extended, arduous process, demanding significant personal investment from the patient [22],[23].

There is a significant evidence base to support both rehabilitative and secondary preventative therapies in stroke [24],[25], but to optimise effectiveness, the issue of nonadherence, and therefore treatment burden, must be addressed. Treatment burden has not been well examined in relation to stroke. Previous syntheses of qualitative studies in stroke have focussed either on the patient experience of illness rather than treatments [26],[27],[28] or on the experience of informal carers [29],[30]. A few syntheses have explored the patient experience of stroke treatments, but these have concentrated either on one specific aspect of the management process [31]–[38] or specifically on the interaction between patient and health care providers [39]–[41]. One synthesis systematically reviewed all qualitative studies of stroke [23] including papers that examined the experience of patients, informal carers, and health professionals, but did not examine the issue of treatment burden. This systematic review therefore seeks to explore the qualitative literature on the patient experience of stroke management with the aim of identifying and describing treatment burden, in order to determine whether treatment burden is an important issue in the context of stroke care. To the best of our knowledge, this is the first such systematic review of qualitative studies of treatment burden in stroke.

## Methods

A protocol was created and the review registered on PROSPERO, the International Prospective Register of Systematic Reviews (CRD42011001123, http://www.crd.york.ac.uk/NIHR_PROSPERO/display_record.asp?ID=CRD42011001123).

### Search Strategy

The review methods have been described in detail elsewhere [42]. A comprehensive search strategy was used to identify qualitative studies seeking to understand the adult patient experience of stroke management. Limitations of English language, year of publication 2000 onwards, and publication in a peer reviewed journal were set. The English language restriction was due to a lack of funding for translation. The year of publication 2000 onwards was chosen to ensure that we collected information about current, rather than historical, patient care. A formal database search strategy using a combination of free text search terms and subject headings was created in consultation with an information scientist; this is shown in Text S1 [42]. Databases searched were Scopus, CINAHL, Embase, Medline, and PsycINFO. The search centred around four main concepts: stroke, treatment burden, patient experience, and qualitative methods. Reference, footnote, and citation tracking were also undertaken. Initially, the search was carried out to include literature published up until March 2011 [42], and this was then subsequently updated to February 2013.

### Inclusion/Exclusion Criteria

We included qualitative studies that explored the adult patient experience of stroke management in any setting (e.g., primary care, secondary care, outpatient, nursing home) and provided information on treatment burden. Full details of inclusion and exclusion criteria for papers are shown in Table S1 and are discussed in detail elsewhere [42].

### Data Screening, Extraction, and Analysis

Title, abstract and full paper screening, data extraction and analysis were undertaken by two individuals with a third party involved for any disagreements. Data extracted for analysis were limited to those describing a range of treatment burdens and to author comments rather than primary data or verbatim quotes. Details of the data extraction instruments developed and used are published elsewhere [42]. Data were analysed using framework synthesis [43],[44] under a coding framework informed by Normalization Process Theory (NPT) [42]. NPT is a robust analytic framework for understanding the organisation and operationalisation of tasks or practices (their implementation), of making them routine elements of everyday life (their embedding), and of sustaining embedded practices in their social contexts (their integration) [45]–[47]. As we are conceptualising treatment burden as a set of practices performed by patients during their chronic disease management that must be implemented, embedded, and sustained in the patient's life, we thought this to be a suitable framework for analysis. NPT has been shown to effectively conceptualise the practices involved for patients during their sickness careers [48], and we have recently shown it to be effective in understanding the treatment burden experienced by chronic heart failure patients [2],[49]. NPT was chosen over a stroke-specific conceptual framework as this enables the possibility of future comparisons between the experiences across a range of chronic diseases and multimorbidity.

During data analysis, data on treatment burden were extracted from the authors' results and discussion sections; each item was then coded independently by two researchers using the coding framework underpinned by NPT (Table S2). This was adapted and refined during data analysis. A careful note was made of any treatment burden that fell outside the coding framework, in order to assess if the framework was ‘fit for purpose’. The data, organised into framework codes, were then examined by the primary researcher, and themes created within and between codes by looking for regularities, irregularities, and relationships between components. As it was felt that data saturation had been reached through our initial search to 2011 which identified 54 papers, for papers identified in our update search to February 2013 one researcher read through the results and discussion sections of each paper and noted any new themes that arose.

A pragmatic approach was then taken to further analyse and reorganise themes into a taxonomy of treatment burden under headings that reflect different processes of stroke care. Themes were then examined in relation to our theoretical framework in order to develop a robust conceptual model of treatment burden in stroke. Several meetings were held between researchers to discuss the emergence of themes and the creation of the taxonomy and conceptual model. We then examined this taxonomy and conceptual model and noted any relationships between components or apparent causal processes, in order to make suggestions for future areas of research or improvements to health service delivery [42].

### Quality Appraisal

Quality appraisal was based upon published guidance by well-known qualitative researchers [50]. The criteria used are shown in Table 1 and detailed elsewhere [42]. Two researchers independently carried out quality appraisal and answers were compared and discussed. Papers were not excluded based on quality appraisal because: (1) our aim was to develop as comprehensive a taxonomy of treatment burden as possible and we intended to minimise the risk of missing any key concepts; and (2) there is currently no consensus on the best way to appraise the quality of qualitative research for inclusion in systematic reviews [51].

**Table 1:** A summary of the quality appraisal of included studies [50].

Appraisal Question	Yes	No	Unclear
Does the research, as reported, illuminate the subjective meaning, actions, and context of those being researched?	68	0	1
Are subjective perceptions and experiences treated as knowledge in their own right?	68	0	1
Is there evidence of adaption and responsiveness of the research design to the circumstances and issues of real-life social settings during the course of the study?	39	27	3
Does the sample produce the type of knowledge necessary to understand the structures and processes within which the individuals or situations are located?	62	3	4
Is the description detailed enough to allow the researcher or reader to interpret the meaning and context of what is being researched?	66	3	0
Are any different sources of knowledge about the same issue compared and contrasted?	38	31	0
Has the researcher rendered transparent the processes by which data were collected, analysed, and presented?	67	2	0
Has the researcher made clear his or her own possible influence on the data?	24	43	2
Is it clear how the research moves from a description of the data, through quotation or examples, to an analysis and interpretation of the meaning and significance of it?	65	4	0
Are claims being made for the generalisability of the findings to either other bodies of knowledge or to other populations or groups?	50	17	2
Is there any other aspect of the study that may affect quality, e.g., conflict of interest?	1	31	37

Each study was appraised using the questions shown in the table. The number of studies with the answers ‘yes’, ‘no’, or ‘unclear’ are shown for each question.

## Results

### Retrieved Studies

**Figure 1:**
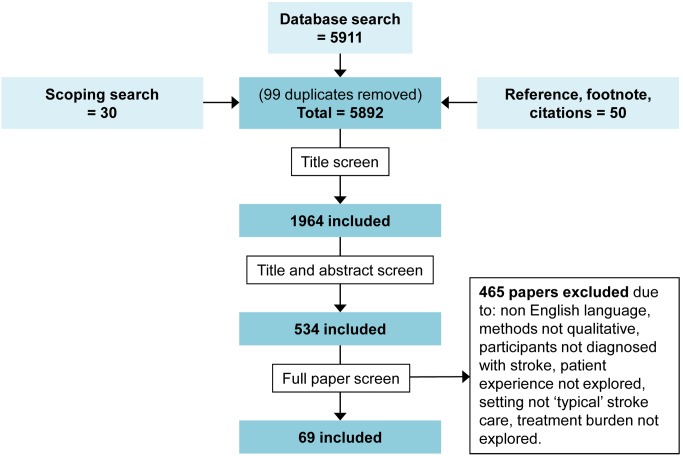
Flowchart demonstrating the screening process of papers in the systematic review. Inclusions and exclusions are shown at each stage.

In total, 5,892 papers were identified, and 69 subsequently met our inclusion criteria. Figure 1 demonstrates the inclusion and exclusion of papers at each stage of the screening process.

### Study Details

None of the included studies stated the investigation of treatment burden as a research objective, but all contained substantial amounts of information on treatment burden in the results or discussion section [42]. Research objectives were noted to vary considerably between studies; to demonstrate this we have broadly categorised papers into the following areas of research (Tables S3 and S4): recovering from stroke, the interaction between patient and health services, return to work/retirement, nursing home experience, coping strategies, living with aphasia, physiotherapy/exercise, motivation/hope in recovery, reintegration into the patient's lifeworld and community, gender differences, the patient's interpretation, returning to driving, multimorbidity, using a wheelchair, eating difficulties, goal setting, and medications. This wide range of categories demonstrates the heterogeneity of included papers.

Key descriptive information of included papers is as follows. Country of study: Sweden (n=19) [52]–[70]; UK (n=12) [7],[71]–[81]; Canada (n=11) [82]–[92]; Australia (n=10) [93]–[102]; US (n=5) [103]–[107]; Norway (n=3) [108]–[110]; Netherlands (n=3) [111]–[113]; New Zealand (n=2) [114],[115]; Iran (n=1) [116]; Republic of Ireland (n=1) [117]; Nigeria (n=1) [118]; and China (n=1) [119]. Participant numbers ranged from 1 to 113. Settings of studies: community (n=34) [7],[52],[55]–[58],[60],[61],[63],[69],[71],[73],[75],[80],[83],[85]–[88],[90]–[94],[96],[99]–[101],[106],[110],[115]–[117],[119]; outpatient (n=5) [54],[62],[65],[74],[118]; care homes (n=3) [111]–[113]; hospital (n=6) [59],[68],[72],[81],[104],[107]; stroke units (n=8) [64],[66],[67],[76],[78],[79],[89],[95]; stroke medical centre (n=1) [108]; and mixed setting such as hospital and community (n=12) [53],[70],[77],[82],[84],[97],[98],[102],[103],[105],[109],[114]. Gender of participants: males and females (n=56) [7],[52],[53],[55]–[57],[59],[60],[62]–[71],[73],[75],[76],[79]–[88],[90],[91],[93]–[100],[102]–[104],[106]–[113],[115]–[117],[119]; male only (n=6) [58],[61],[74],[77],[78],[118]; female only (n=2) [89],[101]; unclear (n=5) [54],[72],[92],[105],[114]. Ages of participants ranged from 22 to 100 years; this was unreported in a few studies (n=7) [53],[54],[72],[105],[106],[110],[114]. Time since stroke (n=41) [7],[52],[55]–[58],[63]–[65],[68]–[71],[74],[78]–[80],[83]–[85],[87]–[89],[91]–[95],[97],[99]–[104],[107],[108],[110],[116]–[118] ranged from under 2 weeks to 16 years. Disability since stroke (n=39) [7],[54]–[56],[58],[61],[63],[65],[67]–[69],[71],[76],[77],[79]–[83],[85],[89]–[92],[95],[96],[99],[100],[102]–[104],[106],[108]–[110],[115],[117]–[119] was very variable. Ethnicity, when reported (n=13), varied between studies [71],[76],[79],[80],[82],[83],[87],[88],[90],[99],[103],[104],[107]. Co-morbidities were seldom mentioned (n=11) [53],[67],[73],[77],[80],[86],[90],[93],[99],[100],[112] nor were medications (n=3) [7],[77],[86]. Qualitative data gathering methods: interviews (n=63)[7],[52]–[74],[76]–[91],[94]–[102],[105],[107]–[119]; focus groups (n=4) [93],[103],[104],[106]; interviews and focus groups (n=2) [75],[92]; additional field notes (n=5) [69],[87],[88],[111],[118]; additional patient observation (n=1) [95]. Data analysis: a variety of qualitative methods were used, and all sought to identify common themes raised by participants (details are described in Table S4). In one study, method of data analysis was not clear (n=1) [104]. Inclusion and exclusion criteria, summary of findings, and study limitations are described in Table S4.

### Quality Appraisal

A summary of the quality appraisal of included studies is shown in Table 1. Papers were generally of a reasonable quality, and aspects of quality that were most poorly demonstrated included acknowledgment of the researchers' influence on the analysis and any note of conflicts of interest.

### Treatment Burden

**Figure 2:**
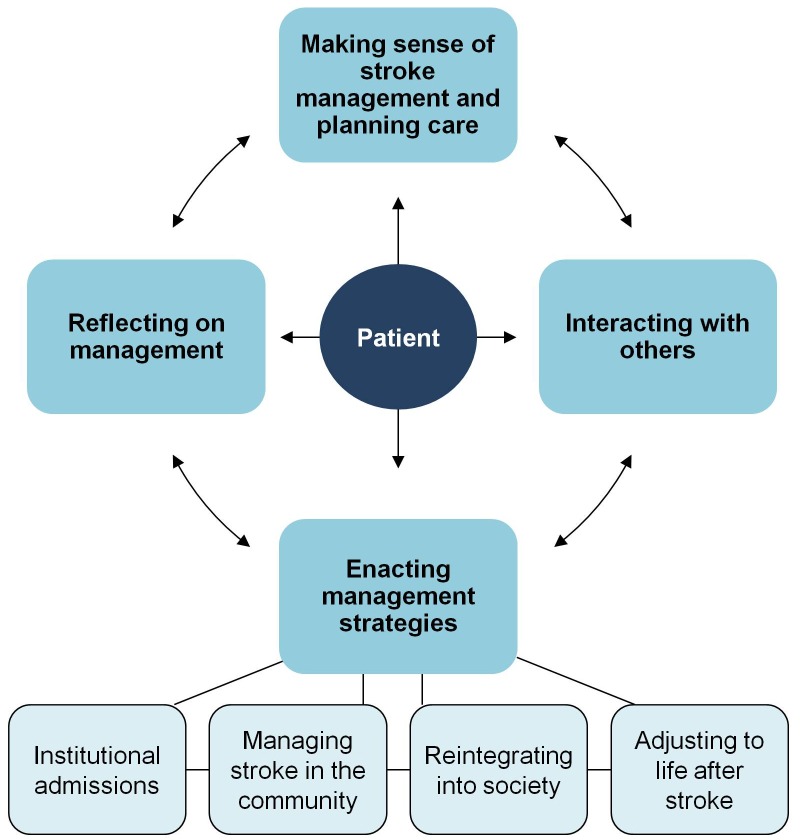
Conceptual model of stroke treatment burden. The arrows represent the possible pathways between components that stroke patients may follow. The ‘enacting management strategies’ component has four subcomponents.

We identified four main areas of treatment burden from the literature: (1) making sense of stroke management and planning care; (2) interacting with others, including health professionals, family, and other stroke patients; (3) enacting management strategies, which includes (a) enduring institutional admissions, (b) managing stroke in the community, (c) reintegrating into society, and (d) adjusting to life after stroke; and (4) reflecting on management. Figure 2 shows a conceptual model of stroke treatment burden. A full taxonomy of treatment burden is shown in Table 2, and a longer version including quotations from included papers is shown in Table S5. No treatment burden was identified that fell outside our coding framework.

**Table 2:** Treatment burden identified from the literature.

Treatment burden category	Taxonomy
(1) Making sense of stroke management and planning care	Making sense of symptoms to aid diagnosis and seek help
	Understanding investigations, acute interventions, medications, risk factor modification, and medical terminology
	Information gathering from health professionals, enduring poor information provision
	Enduring poor information for carers and families from health services
	Carrying out research external to health services
	Understanding the roles of different health professionals
	Working out priorities for rehabilitation
	Goal setting
	Gaining motivation
	Taking responsibility and using initiative, drawing on former life skills
	Managing uncertainty of prognosis
	Problem solving
	Developing coping strategies
	Experiencing negative emotions associated with management strategy, e.g., guilt, frustration
	Using spirituality
(2) Interacting with others	Seeking advice or reassurance from health professionals
	Contacting health professionals for practical help
	Developing relationships with health professionals
	Coping with paternalism from health professionals
	Enduring a lack of understanding from health professionals
	Coping with mismatched ideas about management and recovery with others
	Misdiagnosis at initial presentation
	Having difficulty accessing services
	Experiencing poor communication between services
	Enduring poor continuity of care and consistency of services
	Arranging social care
	Gaining emotional support from friends and family
	Gaining practical support from family and friends
	Experiencing a strain on relationships due to management strategies
	Protecting carers from their burden
	Gaining support from other stroke patients and support groups
	Experiencing stigmatisation due to management of physical disabilities
3) Enacting management strategies	
(3a) Institutional admissions	Undergoing acute care
	Undergoing inpatient rehabilitation
	Fitting into ward routines
	Loss of autonomy and dignity as an inpatient
	Unfamiliar or unpleasant surroundings on the ward
	Admission to a care home
	Learning self-care skills to prepare for discharge
(3b) Managing stroke in the community	Discharge from hospital
	Poor access to services in the community
	Undergoing rehabilitation programmes in the community
	Taking and managing risks during rehabilitation
	Reaching goals
	Establishing and adhering to a medication regime
	Enduring medication side effects
	Managing risk factors
	Adjusting diet
	Managing eating difficulties
	Managing psychological difficulties
	Managing pain
	Regaining communication skills
	Taking physical exercise
	Managing co-morbidities
	Adapting the home environment or finding new accommodation
	Enduring inadequate home services
	Coping with multiple health-related appointments
(3c) Reintegrating into society	Returning to driving or negotiating new methods of transport
	Returning to work
	Acquiring mobility and technical aids
	Negotiating environmental barriers to wheelchair use
	Managing financial difficulties
	Negotiating government benefit systems
(3d) Adjusting to life after stroke	New daily structure to accommodate illness management
	Relearning ways of doing familiar tasks
	Planning activities ahead of time
	Adopting strategies to deal with physical disabilities
	Adopting strategies to deal with cognitive disabilities
	Searching for a sense of self
	Developing acceptance
	Enduring a plateau in recovery
	Changing expectations and examining priorities over the recovery period
(4) Reflecting on management	Decision making about treatments
	Shared decision making about treatments
	Monitoring progress in recovery
	Gauging recovery by comparing self to others
	Self monitoring for further signs of stroke
	Maintaining confidence in care plan
	Keeping up to date with new treatments

A taxonomy of treatment burden in stroke, grouped within categories that correspond to the conceptual model of treatment burden shown in Figure 2.

#### 1) Making sense of stroke management and planning care

During the management of their stroke, patients are required to comprehend a large amount of information [52],[62],[65],[73],[79],[95],[106]. This is an aspect of treatment burden that commonly arises in the literature. Even before diagnosis has been achieved, patients assess symptoms and make decisions about seeking help [65],[92]. They subsequently learn about their diagnosis of stroke, investigations, acute interventions, medications, and risk factor modification [52],[62],[95],[106].

Patients report that they frequently encounter barriers to receiving information from health services in that the provision of information is lacking, inadequate time is allocated, timing is inappropriate, or information is given in a form that is incomprehensible [7],[57],[62],[76],[92],[95],[96],[98]–[100],[119]. One study reports that when asked about their stroke, women are more likely to describe poor information provision from medical staff [62]. Family education also appears to be insufficient, with patients describing how they researched information on behalf of their caregivers due to a lack of available information from health services [106]. The following quotation demonstrates environmental factors described by patients that can prevent the adequate exchange of information:

*(Environmental or contextual) characteristics that resulted in shorter interaction time were the presence of other health professionals or significant others in the room, competing needs of another patient, and health professionals being called away.* [95]

Patients also reported that access to information following the consultation is insufficient [93], resulting in the need for them to spend time seeking information themselves [65],[93],[95],[96],[99] and attempting to make personal sense of the array of changes that are occurring in their lives [52],[72],[78],[88],[95],[106],[108],[111],[112].

Patients often have multiple health care providers, as they interact with a variety of services including health and social care. They describe it as challenging to make sense of and to differentiate between the roles of different individuals and services, for example whom to contact for advice once discharged from hospital [76],[91],[95],[112]. A lack of continuity of care for patients and poor communication between services can result in patients receiving conflicting information from different parties, making it harder to understand the necessary processes that promote recovery [7],[52],[57],[67],[76],[95]:

*A few low motivation patients described some of the stroke unit professionals as giving out unhelpful “mixed messages.” One patient reported that physiotherapists encouraged her to work at rehabilitation. On returning to the ward, however, she thought the nurses discouraged such effort by putting her to bed. This resulted in confusion regarding the correct way to behave.* [76]

Several investigators describe the cognitive processing that patients carry out when managing their stroke. They carefully plan their care, make calculated decisions about their contribution to management, prioritise treatments, and set goals for recovery [53],[54],[58],[67],[70],[74]–[76],[79],[81]–[83],[91],[92],[95]–[98],[100],[110]. Personal goals appear to commonly be focussed on reaching a former social status or role within the family [53],[54],[58],[96],[101]. One study reports that goal setting varies between men and women, with women focussing on resuming tasks within the home and men concentrating on the accomplishment of tasks outside the home environment [59]. Many patients describe a lack of support from health services for this stage and therefore take responsibility and initiative for their own care, drawing on former life skills to plan and organise their recovery [54],[66],[83],[91],[112].

Patients develop expectations of themselves and their health care providers and work at maintaining motivation during the long recovery process [76],[91],[93],[99],[104],[118]. They slowly learn to cope with uncertainty during recovery [75],[93] and problem solve as the need arises [79],[92]. Stroke patients report experiencing emotions associated with stroke management such as frustration at time being taken up by management strategies [68] and guilt at decisions made, for example planning for extended periods of rest during the day [70],[74],[106]. They develop coping strategies to manage emotion such as the use of relaxation techniques, humour, reasoning, positivity, waiting, altruism, and engaging in meaningful activities that give pleasure [56],[66],[68],[72],[78],[81],[93],[96],[102],[103],[107],[115],[118],[119]. Some use spirituality and faith as a method of coping [66],[88],[89],[103],[118],[119].

#### 2) Interacting with others

Along with making sense of stroke and its management, much effort is allocated to engaging with a range of health professionals both in hospital and in the community for emotional support and practical help [7],[57],[62],[65],[74],[81],[89],[92],[93],[102],[106],[118]. One study reports that women are more likely to describe the formation of an alliance with health care assistants on the ward, whist men form alliances with nurses and therapists or other patients [62]. Some patients describe turning to health professionals to validate treatments as appropriate and worthwhile [52], allowing them to take a paternalistic role by relying on their expertise [7],[52],[108],[111],[112]. Others complain about paternalistic care and spend time negotiating with health professionals, preferring a more equal relationship [52],[79],[92],[112]. In one study, men more frequently report trying to exert influence over their care, with women taking a more passive role [62]. Whatever the patient preference, the literature suggests that patients are not adequately consulted about their desires to be involved in decisions about care or about their treatment priorities and goals, and this, along with a lack of information provision, can result in a mismatch in ideas between patients and therapists, leading to frustration for patients [53],[81],[91],[92],[96],[102],[119]:

*All participants with aphasia naturally spoke of the importance of recovering their communicative function. They described intense feelings of frustration, hopelessness, isolation, and depression at not being able to talk. Many stressed that the aphasia was often of higher priority to them than their physical impairments which contrasted with health care systems' focus on physical recovery.* [96]

Many patients report dissatisfaction in their contact with health professionals, complaining of misdiagnosis at initial presentation of stroke [74] and a lack of time and empathy from therapists [57],[58],[66],[75],[79],[81],[99],[102],[103],[111],[118],[119]. It appears that poor interactions between patients and health professionals interferes with the development of trusting relationships, which in turn worsens communication and prevents patients gaining the knowledge they need for the recovery process [7],[56],[57],[62],[92]. It is perhaps not surprising that communication difficulties with health professionals are a particular issue for aphasic patients [58],[96],[115] as the following excerpt illustrates:

*However, he described with contempt how he initially after the stroke had been observed by his therapists. He demanded to be met through dialogues that gave him relevant conversation, support and stimulation. Certainly, he understood the professionals' way of working, but he found it could be done in a more conversational and descriptive way that involved him as a person. The dialogues with professionals should involve him in what was going to happen in spite of his incapability to understand all that was said.* [58]

Patients arrange social care [70],[71],[110] and describe relying heavily on family members for emotional and practical support when managing their stroke [7],[57],[59],[69],[70],[77],[80],[82],[83],[92],[93],[100]–[102],[107],[110]–[112],[115],[118],[119]; one study suggests this is particularly the case for women [59]. Aphasic patients describe using carers to help them with their communication [69],[102]. Such reliance on others can put a strain on relationships as family and friends display overprotection, paternalism, and a lack of understanding about management strategies, and patients experience feelings of guilt about dependency [63],[66],[68]–[71],[75],[78],[80],[90],[91],[103],[110],[115]. Patients describe attempting to protect family from any carer burdens that they may face, for example by arranging respite care [58],[77].

Patients report developing relationships with fellow patients and support groups who provide them with moral support [62],[80],[90],[93],[102],[104],[111],[112],[115], and whom they compare themselves to in order to gauge recovery or validate treatments [56],[64],[66],[67],[69],[76],[78],[80],[88]–[91],[103],[108]. Two papers report that younger, less disabled stroke patients feel uncomfortable attending therapies and support groups alongside older, more disabled patients to whom they struggle to relate [55],[101]. Lastly, enduring stigmatization from others due to the management of disabilities such as the use of a wheelchair or adapted cutlery was reported as a significant treatment burden by patients [52],[56],[63],[71],[74],[80],[88],[99].

#### 3) Enacting management strategies

Enacting work takes many forms and includes the work of enduring institutional admissions, managing stroke in the community, reintegrating into society, and adjusting to life after stroke. We now describe each of these in turn.

#### 3a) Institutional admissions

Stroke patients undergo admission to hospital for acute care [74],[108], then undertake extensive inpatient rehabilitation, attending therapists, taking medications, and working arduously to regain lost functions [53],[54],[57],[77],[81],[82],[102],[103],[108]:

*During initial rehabilitation, the major focus is put on regaining the lost functions. The days are structured around training sessions, be they physical therapy, occupational therapy, speech therapy or ADL training.* [108]

During the rehabilitation process they adjust to their new physical abilities and learn self-care practices to prepare for discharge [70],[81],[92],[111]. They may then be admitted to a care home if discharge into their own home is not feasible [111],[112]. Patients are required to fit into the routines set by institutions [62],[95],[108] and many describe enduring negative environmental circumstances such as unfamiliarity with various gadgets, long waiting times for personal care, inadequate support during mealtimes from staff, poor quality of hospital food, a lack of stimulating activities, and the loss of autonomy, privacy, and dignity whilst on the ward [71],[72],[76],[81],[93],[111]. These complaints were similar in the hospital and nursing home setting, with a particular complaint in nursing homes being a lack of autonomy, with care that is regarded as too paternalistic [111],[112]:

C*are routines, no privacy, time constraints, and lack of familiar activities to perform limit autonomy.* [111]

Patients may receive personal care from hospital staff whilst on the ward, and men report finding this harder to endure than women, describing a feeling of vulnerability. They develop strategies to cope with the situation [59]:

*Men showed vulnerability. They wished to manage by themselves and felt vulnerable when they had to rely on nursing care that involved bodily care. They seemed to have various strategies for dealing with this situation: to accept it or to take command and say how they wanted to be treated. Men described embarrassment at being naked in front of nurses, and also that nurses were sometimes shy of their nude bodies. By conforming to the role of patient the tension could be eased.* [59]

#### 3b) Managing stroke in the community

The transition from inpatient care to the home is an important and often challenging time for patients [70],[111],[112]. Generally, patients report discharge services as poorly co-ordinated, badly managed, and inadequate for preparing patients for life back in the community [52],[70],[71],[74],[82],[91],[93],[111]. Papers from a variety of countries and health care systems describe it as difficult for patients to gain access to advice and services once discharged into the community [52],[71],[91]–[93],[95],[116]:

*One man, who was scared because he suffered a lot from unexpected bodily reactions, wanted to get into contact with his doctor…. He coped with his agony on his own, but he felt abandoned and frustrated. Later on he made the point that attitudes towards handicapped people had changed in general…. He found that he had to struggle with his training and worked at rehabilitation more or less on his own.* [52]

One study carried out in Nigeria reported that even physiotherapy services paid for by patients have inadequate equipment available [118]. Another paper from Canada describes how level of disability affects availability of certain services:

*Although community gyms denied required help for the moderately disabled Mrs C, her impairments were not considered severe enough to qualify for the gym that (severely disabled) Mrs J was able to access. It was difficult for the participants to determine what criteria were in place in each situation and institution. They often learned what disability level qualified them for services in particular settings by trial and error.* [91]

Once home, patients follow routines and integrate management strategies into their everyday lives, for example changing their diet, incorporating physical exercise, and managing risk factors [56],[63],[67],[71],[73],[80],[86],[93],[104],[106]. Patients establish medication regimes and adopt strategies to adhere to these, such as relying on the colours of tablets, using cues as aids, and tying in regimes with daily activities [7]. They endure side effects of medications [7],[81]. They undergo community rehabilitation, striving to achieve the goals that have been set for recovery through hard work and determination [54],[75],[82],[89],[91],[92],[102],[110]. Patients experience a range of environmental risks due to their disabilities and are required to deal with these on a daily basis [99],[92].

Patients are frequently required to acquire equipment and make adaptations to their home to accommodate new disabilities, with one complaint being that new equipment takes up too much space, jeopardising the comfort of their home [68],[70],[71],[77],[80],[87],[88],[90]. Some patients are no longer able to mobilise around their current accommodation, yet waiting times for more suitable housing can be long and arduous [80]. Home care services such as personal care and meal delivery services are described as inadequate, with complaints over both the availability and standard of services, for example the same meal being delivered every day due to dietary restrictions [71],[80],[90].

In this period of time after discharge from inpatient care, the patient schedule is often extremely busy with health care appointments [91],[93],[108], with patients being required to negotiate numerous therapists [74],[77],[91],[108]. As mentioned earlier, poor knowledge about available services, poor access to care, a lack of continuity, and poor communication between therapists are described as frequent and problematic issues [52],[57],[67],[76],[91],[92],[95]:

*Not being given accessibility and continuity pertained to the difficulty of getting in contact with the professionals by telephone and making appointments, delayed appointments with the doctors and physiotherapists, and delays and uncertainties about promised treatments.* [57]

Only one paper discusses the difficulties of managing co-morbidities alongside stroke, with treatments conflicting with one another and predisposing disabilities interfering with rehabilitation [86].

#### 3c) Reintegrating into society

Once home, patients strive to reintegrate into society. Following their stroke, they are usually prohibited to drive for a set period and may be required to take a test set by driving authorities [61]. Many feel frustrated and unsupported by health services as they struggle to understand the logic behind the ban and assessment process, which can lead to rebellion against medical and legal advice with the continuation of driving [61],[82],[100]. Those who can no longer drive are required to negotiate other methods of transport, which can be difficult due to disabilities [80],[100]. It is common for patients to aspire to return to work and regain their former social position, yet describe a lack of support and information from health services as well as friends, family, and work colleagues [54],[101],[107]. They acquire mobility aids for both inside and outside the home, but waiting times can be an issue for the acquisition of such items [77],[87],[88],[90], and some patients describe having to either purchase these themselves or use inappropriate or unsafe aids putting them at risk of falls [80],[100]. The use of wheelchairs was celebrated by many as a way back into society, but environmental barriers such as steps, steep slopes, and narrow doorways were commonly mentioned, although these seem to be less of an issue with powered devices [87],[88],[90],[91].

With regards to financial issues, these are likely to vary from country to country depending on the health care system and welfare provision available [120]. Patients in Nigeria and Iran, both developing countries, describe a lack of rehabilitation facilities for those on low incomes, with poor access to care for those who do not have the means to pay for private services [116],[118]:

*They suffered from having no access to the few existing rehabilitation centres and suffered from low incomes, which made it impossible for them to get such services at their homes. They felt that the government should help them in providing these services as they would then enjoy a better quality of life and escape from physical, emotional and social limitations.* [116]

However, patients in developed countries with government funded health care systems also report suffering financially due to the need to purchase special equipment such as mobility aids and adapted cutlery themselves, or relying on low technology devices due to a lack of economic resources [80],[90]. Patients in developed countries describe the organizations that assist with the arrangement of financial benefits from government agencies as obstructive, poorly co-ordinated, and confusing to navigate [80],[91],[101],[107]. One paper describes how less disabled patients can be denied government benefits, yet be unable to seek employment due to disabilities [101]. Additionally, a fear of losing financial benefits upon return to work due to the inflexibility of government policies can deter patients from returning to employment [91],[107]. One paper gives an example of how conflicting policies can result in significant burden for the patient:

*Mr. D…can walk only 100 yards, but he wants to shop independently for groceries. He asked his doctor to prescribe a battery operated scooter. At the state/provincial level, the health system would pay 80% toward an electric wheelchair, but not for a scooter. Mr. D withdrew the funds from his federal level retirement plan. This money was considered income at the federal government revenue level, and the state/provincial level income supports program for the severely handicapped. He lost income supports until he depletes his retirement funds.* [91]

#### 3d) Adjusting to life after stroke

Following a stroke, patients create a new daily structure to accommodate their new disabilities and treatments [52],[68],[96],[99],[104],[108],[110]. They relearn how to carry out once-familiar tasks [61],[64],[93],[100], and spend extra time planning activities ahead of time [68],[88],[99] as well as adopting strategies to deal with physical and cognitive disabilities, such as taking periods of rest, learning how to get up from a fall, or creating lists or filing systems [56],[58],[69],[70],[75],[83],[88],[93],[99],[102],[110],[115]. Aphasic patients describe using strategies such as carrying communication cards, repeating words, gesturing, and using drawings or technical devices. Some patients, however, found the use of such strategies either inappropriate for their needs or too laborious to use [69].

Following a stroke, patients describe adapting psychologically to their circumstances. They manage this process by searching for a sense of self [64],[66],[70],[78],[87],[90]–[92],[101],[106],[110] and developing acceptance. Acceptance plays a huge part in the recovery process, with patients spending much time and effort working towards and achieving acceptance of their new life that has been altered by stroke and its management [68],[88],[93],[99],[115]. Patients appear initially to be unprepared for the slow pace of recovery, resulting in great disappointment as they meet with unexpected setbacks or a plateau in progress [58],[82],[86],[92],[111],[118], but they describe changing their expectations and priorities over the rehabilitation period as they gain experience of their limitations [58],[64],[68],[70],[82],[90]–[92],[99],[100],[107],[108],[110]:

*Accepting adaptation was felt to represent giving up and relinquishing the struggle to get better. Thus the participants experienced a conflict about whether to develop new habits or not because they associated change with becoming dependent on technical aids, environmental adaptations, and other people. In other words, although adaptation and change seemed to be necessary, they also represented abandoning possible improvements and the hope for independence.* [68]

#### 4) Reflecting on management

Patients must make decisions about their health care, requiring an appraisal of their treatments, either with the help of health care providers [74],[111], or based on their own judgements [7],[56],[58],[65],[81],[99]. Sometimes decisions are made that deliberately contradict advice given by health professionals [7],[54],[58],[71],[99],[111],[118]. This appears often to be the consequence of a breakdown in communication between patient and health professional, or a lack of understanding on behalf of the patient, although informed patient preference is likely to also play a role:

*Discontinuing medication, both prescribed and non-prescription analgesics, was reported by participants in all groups because of insufficient pain relief and side effects or fear of side effects.* [56]

Patients commonly reflect on their achievements and self monitor progress to make judgements about their success [64],[70],[71],[75],[78],[79],[82],[89],[92],[104],[108],[115],[118], comparing their recovery to that of other stroke patients [56],[64],[66],[67],[76],[78],[89],[90] and monitoring for further signs of stroke [70],[75]. Patients describe the need to maintain a confidence in their care plan [7],[66],[70],[79],[82],[89], and one paper described patients keeping up to date with newly available treatments by asking health professionals for information [62].

## Discussion

To the best of our knowledge, this is the first qualitative systematic review to explore treatment burden in stroke. None of the included papers comprehensively covered the entire patient experience of treatment burden; rather each one explored in depth a particular aspect of management or the patient experience in a specific context. Therefore, this review offers a comprehensive taxonomy and conceptual model of treatment burden in stroke. Using this taxonomy, we have been able to examine relationships between components of treatment burden and theorize causal processes. In turn, we shall now make recommendations about areas of health care provision requiring attention from clinicians and policy makers, and areas where further research is required.

A key finding from this review is that stroke patients spend substantial time and effort seeking out, cognitively processing, and reflecting on information about the management of stroke. There is also evidence that the provision of this information by health services is currently inadequate on a global basis. This resonates with previous literature on treatment burden in heart failure patients [2],[49]. It is clear that (1) access to information is poor, (2) time given for the exchange of information is inadequate, (3) the information given is not easily understood by patients and is not tailored to suit their needs, and (4) information is often given at times when patients are not able to process it. These four factors result in patients feeling poorly informed and consequently expending time and energy on researching their stroke management. Both communication during the clinical encounter and provision of information to patients must be improved by health services, as patients' understanding of the rationale behind therapies and their trust in management plans is pertinent to achieving optimum adherence [7]. Knowledge deficits mean patients are ill equipped to plan and organise their care, to develop coping strategies, and to set goals for recovery. The clinical implications of this knowledge deficit require further exploration. A recent Cochrane Review concluded that improved information provision to stroke patients showed no improvement in health-related behaviours, health service usage, or mortality. However, the review did demonstrate an improvement in patient knowledge (which could arguably lead to more informed decision making), increased patient satisfaction, and a small reduction in depression. It also suggested that interventions that actively involve the patient and carers with planned follow-up for reinforcement had a better effect on mood. The authors concluded that the best way to provide information is still unclear, and this needs further investigation [121]. We hypothesise that improved information provision as part of a more comprehensive intervention to decrease treatment burden on a wider level may be more effective, and this should be explored through both quantitative and qualitative research.

In addition to poor provision of information by health services, the exchange of information between patient and professional generally appears to be substandard, resulting in a mismatch in ideas regarding goals and care preferences. This leads to patient dissatisfaction, a prerequisite for nonadherence to subsequent management plans, as confidence and motivation are negatively affected [7]. It is therefore vital that health professionals spend time with patients to gauge their care preferences. Previous research has shown that during the consultation, patients are not always forthcoming with their own agendas [122]; therefore, eliciting their ideas, concerns, and expectations is an important skill on the part of the health professional, and one that requires to be learned and practiced. Additionally, busy clinics and ward rounds can bestow time constraints that hinder communication. Research aimed at improving communication must therefore include both patients and health professionals at the consultation level to achieve pragmatic interventions. Health service reconfiguration must prioritise enhanced communication between clinician and patient, with outcomes such as treatment burden, patient satisfaction, treatment adherence, and mortality being monitored.

The organisation of services at both macro and micro levels appears to significantly affect treatment burden. The papers in this review describe interactions between stroke patients and a variety of professionals including hospital doctors, nurses, general practitioners, speech and language therapists, physiotherapists, occupational therapists, and social workers. Because of the long-term nature of stroke rehabilitation, patients describe the importance of developing relationships with their therapists, but this is made difficult by poor continuity of care, in both the hospital and acute setting. Patients describe receiving ‘mixed messages’ from different carers who do not communicate with one another. Health professionals must establish good methods of communication with each other and provide individualised, holistic, patient-centred care. If case meetings cannot be carried out face to face then adequate secure methods of communication such as clinical email systems must be utilised.

These findings appear to resonate across various countries in our review; however, issues such as poor continuity of care are likely to depend on organisation of health care systems, which may vary substantially between countries and localities. Some services, for example, are available through government funded initiatives and others require payment at point of care, and the standard of these services are likely to vary considerably [120]. It would therefore be pertinent for future research to examine differences in stroke care provision between localities and any resultant effects on treatment burden. Research can then inform changes to practice and policy at a local level. Additionally, the use of certain technologies may be less available in low-income countries, so guidelines must take account of this.

Attending and planning appointments takes considerable time and effort from the patient, made all the more difficult by poorly organised, fragmented services. Patients are also required to manage often complicated medication regimes and endure any side effects. In westernised countries, patient care has moved away from being patient centred with subspecialisation of therapies and a focus on therapist- rather than patient-set goals [53]. As well as having an effect on treatment burden due to sheer volume of appointments and medications, therapies can contradict or interfere with each other and cause difficulties for patients. This is particularly relevant for stroke patients with multimorbidity who additionally have other treatment regimes to deal with simultaneously [123]. Any measurement of treatment burden developed must be able to take account of multimorbidity to truly reflect the burden experienced by patients. Appointments should be allocated in consultation with the patient as much as possible, with evidence-based strategies such as reminder systems being utilised to improve attendance [124].

Another important treatment burden relates specifically to hospitalisation experiences. The hospital stay itself is frequently described by patients as unpleasant, with a lack of autonomy over treatments and loss of control over daily routines. Again, this is likely to vary significantly between localities. In this review, stroke patients describe spending long periods of time on rehabilitation wards feeling understimulated and bored. Younger patients describe a lack of tailoring of rehabilitation services to suit their needs. Such issues should be addressed by health care providers, particularly as initial results of recent randomised control trials have shown improved functional recovery associated with very early mobilisation following stroke [125],[126]. Improved communication between staff and patients would allow for patient autonomy, and recreational activities or time off the ward should be available to patients, in order to boost morale and maintain motivation.

In the community, social care systems such as home helps and meal delivery systems are described as being of a very poor standard by patients, for example providing a very narrow range of food at inconvenient times of day. The provision of personal care such as help with showering also appears to be lacking. Improvements to these services are vital for adequate patient care as they provide the fundamental aspects of human functioning. Further qualitative work is required to explore these services in different localities, as information concerning this was limited within this review.

Patients describe having difficulty accessing care both as an inpatient and in the community. This resonates across both developed and developing countries in our review. Patients feel that time with therapists is too short, mirroring the lack of time spent imparting information as discussed above. Clinicians must ensure that time is available for consultations with patients. Although this may cost health services money in the short term, it will prevent nonadherence and therefore wasted expenditure in the longer term [127].

Discharge from hospital is described as a particularly difficult time for patients, with a sense of abandonment without adequate preparation. Patients feel that services are terminated prematurely, and they feel uncertain whom to contact should they need help and advice. This is a very important step in the recovery process, and discharge should be timed appropriately so that services are in place and patients are armed with the appropriate information. Disabled patients need to acquire technical aids and make adaptations to the house, or to move to more appropriate accommodation, and should be supported as much as possible during this time. A point of continuous contact such as a stroke liaison nurse can improve patient satisfaction and support the process of discharge and community rehabilitation [128]. Reintegrating into society, regaining driving ability, and employment are important steps in recovery, and patients need access to appropriate services for support.

Financial difficulties due to stroke management seemed to arise for patients in both developing and developed countries, although only two papers from developing countries were found, so this requires further exploration. In the developing countries, access to care appears to depend on the ability to self-fund therapies, whilst in countries with universal health care access, difficulties can arise when negotiating complicated systems; patients also often self-fund as a result of this.

Several papers discussed the psychological difficulties patients encounter during the recovery process, yet access to psychological therapy seems to be scarce. Patients describe spending time reflecting on their progress, adjusting to their new circumstances, and maintaining motivation. Better access to counselling or psychology services is therefore pertinent for stroke patients.

Two studies made gender comparisons of the patient experience of managing stroke [59],[62], but little information was provided to allow comparisons based on other patient characteristics such as age, ethnicity, and socio-economic deprivation. Differences, if any, associated with such patient characteristics should be considered further, especially in relation to the development of any patient-reported outcome measure of treatment burden.

For the first time, our study approaches the management of stroke as a global set of practices carried out by patients in multiple contexts. The extent of treatment burden can be affected not only by the nature of illness but also by the micro- and macro-organisation of health services. We hypothesise that the components of treatment burden can amalgamate [12], and if treatment burden exceeds patient capacity, then nonadherence may occur [1], a problem in chronic disease management well recognised by the World Health Organisation but not yet fully understood [13]. We therefore propose that to improve patient adherence, we must address the organisation and delivery of health services to minimise burden on patients.

### Aspects of Treatment Burden Missing from the Literature

Certain aspects of stroke management were mentioned less often than anticipated: the process of acute care, medications, social care, the stroke liaison nurse, and the use of new technologies. Only one study addressed multimorbidity [86], although it is known that patients with stroke often suffer from multiple morbidities [129], each with its own management plan and demands that may interfere with one another. Further primary studies are required that explore these aspects of stroke management and the treatment burden that may arise for patients.

### How This fits In with Current Knowledge

In comparison to our recent work on treatment burden in heart failure patients, there was less information available on the burden of medications, particularly polypharmacy, side effects, collecting prescriptions, altering routines as required, and drug interactions [2],[49]. More emphasis was placed by stroke patients on the development of coping strategies and goals for rehabilitation, and on adjusting to life after the illness has presented. These changes may be due to differences in methodologies, or they may reflect the differences in onset and management of these two chronic diseases.

However, one important similarity between this review and previous work with heart failure patients is that the care of patients with both chronic diseases is very sensitive to investments in service provision. Shifts from intensive care environments, such as rehabilitation centres, to self-help and community-based services may fundamentally change the burden of treatment from professionals to patients and caregivers. As our review documents, patients and caregivers are already burdened and perceive they gain inadequate support from health care services. Further work toward understanding how policy changes in health care affect the balance of burden and capacity for patients and caregivers is essential to assess these dynamic interactions.

A recent systematic review explored the conceptualization of treatment burden in chronic disease. This review examined attributes, antecedents, and consequences of treatment burden [130] but differs to ours in that most studies included were quantitative and cover a wide range of chronic diseases without differentiating between these in the results. An interesting overview of the concepts of treatment burden is therefore described, rather than a deeper exploration into disease-specific aspects of treatment burden.

A recent paper by Eton et al [3] created a conceptual framework of treatment burden in patients with complex chronic disease that resonates highly with our results. Eton et al. carried out their study in the US where patients are required to negotiate with insurers and face financial challenges that are perhaps more profound than in countries with a universal health care system. The financial implications of chronic disease management for patients in differing countries appear to be poorly examined elsewhere in the literature, and this requires further exploration.

Another recent paper, by Tran et al. [4], sought to develop a method of measurement of treatment burden in multimorbid patients. Although there were many similarities to our results, two differences to highlight are that the measurement developed did not include information on making sense of treatments, as acknowledged by the authors, and the impact of health care organisation was not explicitly explored. Medication side effects were not included in the instrument because of the conceptual nature of the study, and financial implications did not arise in patient interviews, because of the universal health care system in France where the study was conducted.

### Limitations/Strengths

The search was limited to publications from the year 2000 and onwards. This date was chosen because our review is aimed at understanding current, rather than historical, patient experiences of stroke in order to inform current clinical practice and policy. Global management of stroke has changed in recent years with the introduction of stroke units and community rehabilitation programmes [120],[131], and hence we believe this to be justifiable, but appreciate that it could be viewed as a limitation. We restricted our search to English-language papers, but no geographical restriction was set, and our review includes papers from a variety of countries. However, the language restriction may have imposed a degree of geographical restriction, and there was a paucity of data from low-income countries. The exclusion of methodologies such as telephone and postal questionnaires could be regarded as a limitation, as some studies exploring treatment burden may have used these methods. Similarly, grey literature was excluded to manage the scope of the review.

Important strengths of our review are that we conducted an exhaustive search and our tight inclusion criteria allowed us to avoid collecting too broad a spectrum of methodologies, as high numbers of studies using extremely varied methods made in-depth analysis of the data and applicability of findings extremely challenging. Our approach helped us to maintain focus whilst producing a rich picture of stroke management. As a result, the number of studies included was considerable yet still feasible for the application of qualitative analysis. Finally, a particularly novel aspect of this review was our approach to data analysis using a coding framework underpinned by a robust theory, NPT. The use of framework synthesis [42] was appropriate as we had a preconceived research objective based on our knowledge of the literature and clinical experience, yet this method ensured that our results arose directly from the data. We found this approach highly pragmatic and useful, as have others, [44],[132] and believe it enhanced transparency of coding. While the suppression of interpretive creativity [133] is a potential risk, we attempted to minimise this by paying close attention to any data that may have fallen outside the framework, and iteratively adapting the framework during analysis to ensure that analysis was somewhat inductive [42]. We found this novel method of data analysis very useful for identifying the components of treatment burden in stroke from the patient perspective, and did not find any aspects of treatment burden that fell outside this framework.

The large variation in research objectives of included studies means that a diverse range of treatment burdens are described. A major advantage to our review is that it pieces together information about treatment burden from various sources to create a more comprehensive picture than is usual for this type of study. However, one limitation is that the papers and therefore participants studied were heterogeneous, making comparisons between papers difficult, for example to compare papers from different countries. It is likely that there is significant variation in health system delivery between countries, including availability of services through state-sponsored insurance.

Both severity of stroke and level of disability are likely to influence treatment burden, yet both are generally poorly described in the included papers, and those that do describe them use varying measures and terminology. It could be argued that the most physically and mentally impaired may be the most burdened and the least likely to participate in research, a common problem in the research arena. For example, the papers that study aphasic patients describe a particular difficulty for these patients in communicating with therapists and carers, a perhaps unsurprising but important finding [58],[96],[115]. The inclusion of papers that study aphasic patients and wheelchair users is almost certainly a strength of our review [58],[87],[88],[96],[115], but there is likely to be an over-representation of able-bodied patients. Time since diagnosis is also likely to influence treatment burden, as patients adjust to their condition and the process of rehabilitation. Interestingly, our quality appraisal instrument did not judge quality based on the detailed provision of patient characteristics. During appraisal, judgements were made about whether the sample was appropriate for the research objectives of that individual study, and if authors assessed generalisability accurately. In the qualitative research arena, focussing on these factors tends to be more pertinent than producing work that is generalisable to other populations, one argument made by those against qualitative syntheses [134]. We believe, however, that with transparency in reporting about generalisability, qualitative synthesis is invaluable for informing clinical practice and health policy.

### Conclusion

We have created a comprehensive taxonomy of treatment burden underpinned by international research which has the potential to drive service improvement. The aim of this review is not to produce a taxonomy that is universally generalisable, but one that gives insight into the scope of burdens experienced by patients and can inform the development of measures and interventions. Our taxonomy suggests that treatment burden in stroke can be broadly categorised into: (1) making sense of stroke management and planning care, (2) interacting with others, (3) enacting management strategies, and 4) reflecting on management. Patients describe care as fragmented and lacking in continuity, with poor communication between patient and clinician and between health care providers. Information provision is generally poor, and patients would like clinicians to spend more time with them. There is considerable room for improvement in both inpatient and community services.

Treatment burden appears to be greatly affected by the micro and macro organisation of health services, which is likely to vary considerably between localities. Further work is recommended to better understand the patient experience of treatment burden in stroke in varying contexts and to explore how it may vary by patient demographic or clinical characteristics. Treatment burden should be investigated in relation to other chronic diseases, and importantly in patients with multiple morbidities. This could inform the generation of a patient-reported outcome measure to be utilised by both policy makers and health care providers, and could serve as a new goal for quality improvement.

## Supporting Information

Figure S1 **PRISMA flowchart.**
(DOC)

Table S1 **Inclusion and exclusion criteria for papers.** The inclusion and exclusion criteria used during the screening process.
(DOC)

Table S2 **Coding framework informed by Normalization Process Theory.** The framework used to code data from each included paper.
(DOCX)

Table S3 **Participant details.** Details of participants in each included study.
(XLSX)

Table S4 **Study methods and results.** Details of research methods and results for each included study.
(XLSX)

Table S5 **Taxonomy of treatment burden with exemplar quotations.** A taxonomy of treatment burden in stroke as shown in Table 2, with the addition of examples of quotations from included studies.
(DOC)

Text S1 **Search strategy.** Details of the strategies employed for searching Medline, Embase, PsycINFO, CINAHL, and Scopus databases.
(DOC)

Text S2 **PRISMA statement.**
(DOC)

## Abbreviations

NPT: Normalization Process Theory
