# Supplementary material for: Uncovering Treatment Burden as a Key Concept for Stroke Care: A Systematic Review of Qualitative Research
Source: PLoS Med. 2013 Jun 25;10(6):e1001473. doi: 10.1371/journal.pmed.1001473 (PMC3692487; doi:10.1371/journal.pmed.1001473)
Supplement: Figure S1 — PRISMA flowchart. (DOC) [file pmed.1001473.s001.doc]

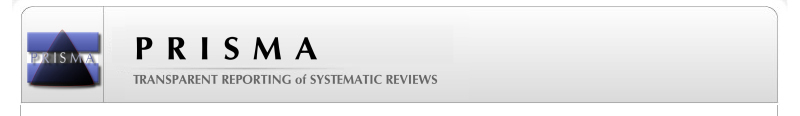
**PRISMA 2009 Flow Diagram**

**Screening**

**Included**

**Eligibility**

**Identification**

Records identified through database searching
(n =5911)

Additional records identified through other sources
(n = 80 )

Records after (n=99) duplicates removed
(n = 5892 )

Records screened
(n = 5892)

Records excluded
(n = 5358)

Full-text articles assessed for eligibility
(n = 534)

Full-text articles excluded,
(n = 465)

Due to: non English language, methods not qualitative, participants not diagnosed with stroke, patient experience not explored, setting not ‘typical’ stroke care, treatment burden not explored.

Studies included in qualitative synthesis
(n = 69 )
