# Supplementary material for: Uncovering Treatment Burden as a Key Concept for Stroke Care: A Systematic Review of Qualitative Research
Source: PLoS Med. 2013 Jun 25;10(6):e1001473. doi: 10.1371/journal.pmed.1001473 (PMC3692487; doi:10.1371/journal.pmed.1001473)
Supplement: Table S1 — Inclusion and exclusion criteria for papers. The inclusion and exclusion criteria used during the screening process. (DOC) [file pmed.1001473.s002.doc]

Table S1 - Inclusion and exclusion criteria for papers.

*The inclusion and exclusion criteria used during the screening process.*

| **Inclusion Criteria** |  |
| --- | --- |
| Types of studies | Publication date 2000 (inclusive)-present. |
|  | Studies from any geographical location. |
|  | English language. |
|  | Studies using qualitative methods of analysis (to describe patterns or themes raised by participants) seeking to understand the patient experience through direct contact with patients or direct observation, describing the processes of illness management experienced by patients and their active contributions towards this. This includes original qualitative studies, studies involving secondary qualitative analysis of qualitative data, and a qualitative study as part of a mixed methods study e.g. the study also has a quantitative component. |
| Types of participants | Adults (>18 yrs). |
|  | Diagnosed with at least one CVA /CVD / stroke, including ischaemic, intracerebral haemorrhage or subarachnoid haemorrhage, any anatomic location, as per WHO’s definition ‘rapidly developing clinical signs of focal (at times global) disturbance of cerebral function, lasting more than 24 hours or leading to death with no apparent cause other than that of vascular origin.’ |
|  | Being treated in any ‘usual care’ setting: primary; secondary; tertiary care, e.g. in the hospital, hospice, community, home or rehabilitation. |
|  | Receiving care typical for that geographical location i.e. not part of experimental or pilot studies. |
| Types of outcome measures | Logistical burdens e.g. interacting with health care organisations, carrying out administrative work involving insurance companies or others, organising appointments or visits from health professionals, organising rehabilitation, arranging transport, funding care. |
|  | Technical burdens e.g. enacting lifestyle changes, performing rehabilitation exercises, modifying environments, taking medications, using assistive technologies. |
|  | Relational burdens e.g. enrolling family, friends and health professionals for support, initiating interactions with possible carers and supporters, maintaining relationships during rehabilitation. |
|  | Sense making burdens e.g. conceptualising problems, understanding and learning about management strategies, knowing when to seek help, differentiating between treatments. |

| **Exclusion Criteria** |  |
| --- | --- |
| Types of studies | Non English language. |
|  | Published pre 2000. |
|  | Grey literature / not published in a peer reviewed journal. |
|  | Dissertations /theses. |
|  | Proceedings. |
|  | Published abstracts. |
|  | Studies using the following methodologies: Lexical studies that analyse natural language data presented as quantitative results; studies without a sampling procedure; qualitative studies using questionnaires or other methods that do not involve direct contact or observation of participants; studies describing the patient experience in an experimental situation rather than ‘typical’, e.g. during pilot schemes or intervention/experimental trials; any study where qualitative data not analysed i.e. uninterpreted data; case report; any review (systematic, narrative, qualitative); any quantitative study (RCT, non RCT, observational, cohort, case control). |
|  | Treatment guidelines documents. |
|  | Commentary articles, written to convey opinion or stimulate research /discussion, with no research component. |
| Types of participants | Children (<18 yrs). |
|  | No CVA diagnosis. |
|  | Diagnosis of TIA, subdural haematomas, infarction / haemorrhage due to infection or tumour, cerebral palsy or any other neurological deficit. |
|  | Mixed groups of participants e.g. patients and carers or health care providers, unless results from patients are explicitly separate from other participants. |
| Types of outcome measures | Anyone other than the patient’s perspective of patient work e.g. health professionals. |
|  | Burden on health services / systems or health professionals. |
|  | Economic burden at a society level e.g. costs to government or councils. |
|  | Carer work or burden. |
|  | Standardized, patient-reported measures of treatment burden or illness burden / activities of daily living / quality of life / functional capacity. |
