# Supplementary material for: Uncovering Treatment Burden as a Key Concept for Stroke Care: A Systematic Review of Qualitative Research
Source: PLoS Med. 2013 Jun 25;10(6):e1001473. doi: 10.1371/journal.pmed.1001473 (PMC3692487; doi:10.1371/journal.pmed.1001473)
Supplement: Table S2 — Coding framework informed by Normalization Process Theory. The framework used to code data from each included paper. (DOCX) [file pmed.1001473.s003.docx]

Table S2 – Coding framework informed by Normalization Process Theory.

*The framework used to code data from each included paper.*

| **Coherence**  ***(Sense-making work )***  **Understanding the prospect of having a stroke, what this means and how the condition may be managed.** | **Cognitive Participation**  ***(Relationship work*)**  **Investing personal and interpersonal commitment to living with the condition and its management.** | **Collective Action**  ***(Enacting work)***  **Investing effort and resources in management, carrying out tasks.** | **Reflexive monitoring**  ***(Appraisal work)***  **Reflecting on the effects of therapies in retrospect and determining whether to modify them.** |
| --- | --- | --- | --- |
| **Differentiation**  Understanding and differentiating between risk factors, investigations, treatments and the roles of different health professionals or services. Prioritising treatments and activities. | **Enrolment**  Engaging with friends, family and health professionals with regards to diagnosis and illness management to enable them to provide support. Adjusting relationships to accommodate new roles as a result of illness management. Protecting family members during management. | **Skill set workability**  Setting a routine/strategy to cope with symptoms, exacerbations, and emergency situations i.e. therapeutic interventions. Enacting activities with a view to achieving goals. Learning through practice. Controlling risks associated with recovery. | **Reconfiguration**  Altering a set routine when required such as medication regimes or appointments, to fit in with daily activities or other arrangements. Learning a new way of doing things after stroke. Altering priorities and ways of thinking due to stroke management. |
| **Communal specification**  Gaining information about illness management with the help of others, for example friends, family or health professionals. Receiving diagnosis, or misdiagnosis. | **Activation**  Arranging help (e.g. logistical, administrative, or expert) from health professionals, social services or friends and family. | **Contextual Integration**  Making sure you have the right financial and social resources, and integrating the illness into social circumstances. Managing potential environmental dangers through making resources available. Experiencing good or bad environmental situations. Adjusting to new social role in society or life circumstances such as unemployment. | **Communal Appraisal**  Discussing or altering current management plans already initiated, in discussion with health professionals or friends and family. Recalling previous events with friends and family. |
| **Individual specification**  Achieving your own understanding of illness management in personal terms, through personal research such as reading, or personal life experience. | **Initiation**  Using organisational skills to arrange one’s own contribution to management, such as arranging prescriptions, social care and transport to appointments. | **Interactional workability**  Taking treatments, enacting lifestyle changes, attending appointments, enduring side effects. Enduring poor health care or care that does not meet expectations (e.g. poor interactions). Enduring setbacks in recovery. Learning self care. The work of rehabilitation. The work after discharge. Enduring intrusions and interventions from family members, including negative interactions such as a lack of support. | **Individual appraisal**  Assessing individually whether to continue or alter current management plans. Recalling previous events. Monitoring symptoms and progress (but not as a routine, see below). |
| **Internalization**  Relating your own experiences to illness management, understanding any implications. Knowing when to seek help. Understanding one’s own contribution to reducing risk, knowing limitations and risks due to stroke. Calculating safety risks. Maintaining motivation and determination. Overcoming barriers in receiving information. Developing expectations of health services. Making sense of progress in recovery and one’s own contributions to this. Setting goals for recovery. | **Legitimation**  Seeking reassurance about treatments from others about appropriateness of management plans. Gaining confidence in the success of treatments from others. Dealing with stigmatisation or a mismatch in ideas and expectation from others. Reaching an understanding that treatments are’ the right thing to do’. Comparing yourself to others to validate treatments. | **Relational Integration**  Maintaining confidence in health professionals and their interaction with each other. Maintaining confidence in care plan. Coping with multiple caregivers. Enduring system failures caused by poor communication / interaction between service providers. | **Systematization**  Developing ways of keeping up to date with newly available treatments. Routine self monitoring. |
