# Supplementary material for: Uncovering Treatment Burden as a Key Concept for Stroke Care: A Systematic Review of Qualitative Research
Source: PLoS Med. 2013 Jun 25;10(6):e1001473. doi: 10.1371/journal.pmed.1001473 (PMC3692487; doi:10.1371/journal.pmed.1001473)
Supplement: Table S5 — Taxonomy of treatment burden with exemplar quotations. A taxonomy of treatment burden in stroke as shown in Table 2, with the addition of examples of quotations from included studies. (DOC) [file pmed.1001473.s006.doc]

| 1) Making sense of stroke management and planning care  Table S5 – Taxonomy of treatment burden with exemplar quotations.  *The taxonomy of treatment burden in stroke as shown in Table 2, with the addition of examples of quotations from included studies.* | Making sense of symptoms to aid diagnosis and seek help | The patients ignored the first symptoms of neurological damage although they all were in places where they could easily get in contact with a qualified hospital. M2 was attending a conference. F1 was going to have her car checked. M1 was at his country residence near the town [65]. |
| --- | --- | --- |
|  | Understanding investigations, acute interventions, medications, risk factor modification and negotiating medical terminology | Although the stroke was understood as a serious disease, which they wanted to prevent, they did not seem to be aware of which risk factors were relevant. At least they did not bother to minimize these risk factors [53]. |
|  | Information gathering from health professionals, enduring poor information provision | Not being adequately informed concerned what the participants described as absent, contradictory or incomprehensible information. Some of them had not received any information other than what was given to them in a brochure about stroke. Others had wanted more detailed information about their brain injury, the reason for examinations performed, the results and the prognosis. Further, contradictory information with regard to the cause of their stroke and about their treatment was described [57]. |
|  | Enduring poor information for carers and families from health services | Another perspective on this theme was the call for family education. Participants felt that their families required more information about fatigue, particularly regarding the long-term nature of the problem [106]. |
|  | Carrying out research external to health services | Often, the health care system was unable to offer these patients a doctor who fulfilled their demand of competence and behaviour. Instead, they picked information from sources at hand, for instance books and friends [65]. |
|  | Understanding the roles of different health professionals | As a result of all the changes, patients feel insecure on admission, describe themselves as laypersons and the health professionals as experts, and find it difficult to judge the work of the health professionals [112]. |
|  | Working out priorities for rehabilitation | Participants with communication difficulties described these as overwhelming and many became preoccupied with regaining their communication abilities, to the extent that other needs or physical symptoms were ignored [94]. |
|  | Goal setting | Although the major focus is on bodily improvement, the patient also directs attention towards more practical issues. In this phase, the major concern is related to the possibilities of returning home. The patient initiates an appraisal process of what problems he might face and possible ways of handling them [108]. |
|  | Gaining motivation | Managing and maintaining motivation for rehabilitation regimes was difficult for some participants. Some who were unable to muster motivation for an extended time appeared to be hampered by a lack of acceptance about what had happened to them [93]. |
|  | Taking responsibility and using initiative, drawing on former life skills | When the informants experienced that the rehabilitation professionals did not take action, they took control of the situation themselves. The informants expressed pride in their own capacity to take the initiative and in their ability to take action [54]. |
|  | Managing uncertainty of prognosis | Uncertainty about prognosis, along with anxiety about the degree of recovery that would be achieved became predominant concerns during rehabilitation. For some participants, distress arose from overly optimistic expectations about speed of recovery; others perceived that there was some deadline by which they must recover [93]. |
|  | Problem solving | One way in which participants perceived therapy helped to facilitate progress was by responding to problems and solving each difficulty as it arose [79]. |
|  | Developing coping strategies | It is clear from the discussion that patients saw physical limitations as challenges to be overcome by their own individual effort. They believed that it was their job to recover from the physical consequences of stroke, and they developed personal coping strategies in order to do so [103]. |
|  | Experiencing negative emotions associated with management strategy e.g. guilt, frustration | Many participants acknowledged that napping during the day was part of their daily routine, although they often expressed feeling guilt about it [106]. |
|  | Using spirituality | Spiritual and religious resources were also mentioned by four people as helping them deal with the difficulties in their post-stroke lives [85]. |
| 2) Interacting with others | Seeking advice or reassurance from health professionals | The judgements made by the health care providers about the recovery are important because the stroke survivors use them to validate the progress of their rehabilitation [52]. |
|  | Contacting health professionals for practical help | Temporary pain relief was obtained from prescribed medication, which they received after contacting the doctor or nurse, so they were able to get some sleep [57]. |
|  | Developing relationships with health professionals | To establish a personal relationship with the staff was useful in several ways. They felt more free to ask for help with personal care and to ask questions about their diagnosis and prognosis [62]. |
|  | Coping with paternalism from health professionals | Although participants perceived therapy to be an important factor, five participants suggested an inequality within the relationship. They described feeling controlled by the therapists regarding which direction therapy would take and the activities which were to be practiced. Not all participants were comfortable with what they perceived to be a passive role [79]. |
|  | Enduring a lack of understanding from health professionals | Lack of understanding had also to do with attitudes, with what they experienced as unreasonable demands on the part of both the professionals and their next of kin. They described incidents where the professionals had been annoyed with them when they had been in a bad mood or very tired during their physical exercises, or when encountering over-strained professionals. There were also experiences of a feeling of superiority on the part of the professionals, expressed in a comment or a glare [57]. |
|  | Coping with a mismatch in ideas about management and recovery with others | For them recovery involved dimensions that were not included in the health care professionals’ concept….The goal for them was either to recapture their former social position or to adapt to another life situation [55]. |
|  | Misdiagnosis at initial presentation | Several participants reported having multiple contacts with primary care services prior to diagnosis of stroke and hospital admission [74]. |
|  | Having difficulty accessing services | Patients feel that therapy and supervised exercises in the ward facilitate regaining self-care, but they experience a lack of therapy and supervision, for example, when their therapist is ill or during weekends. In the patients’ view, this problem can be solved but patients find it difficult to judge [112]. |
|  | Experiencing poor communication between services | Some hospital procedures (i.e., ordering medical tests, waiting on transfer from another ward) that involved other hospital departments created uncertainty and made it difficult for some participants to be given information on some topics (i.e., which tests they would be having on a certain day and at what time) [95]. |
|  | Enduring poor continuity of care and consistency of services | Messages—A few low motivation patients described some of the stroke unit professionals as giving out unhelpful “mixed messages.” One patient reported that physiotherapists encouraged her to work at rehabilitation. On returning to the ward, however, she thought the nurses discouraged such effort by putting her to bed. This resulted in confusion regarding the correct way to behave [76]. |
|  | Arranging social care | Home care staff shopped for two participants; this worked well for one after some initial misunderstandings [71]. |
|  | Gaining emotional support from friends and family | Meaningful relationships – Support: “Don’t do it alone.” Overwhelmingly, participants highlighted that the support offered through meaningful relationships was a key component contributing to living successfully with aphasia [115]. |
|  | Gaining practical support from family and friends | Being given practical and emotional support by their next of kin was experienced when they received help and the next of kin had performed certain tasks for them. They described help from their spouses in specific activities of daily living, and a few described getting support in their daily exercises. They appreciated being taken out for a drive or a walk. Emotional support was also experienced in connection with being together, mainly with one’s spouse. Further there was the concern shown by the next of kin by keeping in contact regularly [57]. |
|  | Experiencing a strain on relationships due to management strategies | Negative supports hindered strategies designed to adapt to disabilities. For example, one woman’s efforts to adapt to her cognitive disabilities and enhance her sense of mastery and control were repeatedly foiled by her husband’s negative attitudes and attempts to remove her adaptive aids [83]. |
|  | Protecting carers from their burden | He was reluctant to go out with his wife, feeling that he was too much of a burden. He did, however, persuade a friend to take him to the local pub once a week in his wheelchair. He felt that this was his way of giving his wife a rest from coping with his stroke [77]. |
|  | Gaining support from other stroke patients and support groups | Participants described the unique and important role played by stroke support groups. As well as providing an enjoyable social gathering, participants described feeling understood by others in the group, in a way that family or friends could not understand [94]. |
|  | Experiencing stigmatisation due to management of physical disabilities | Some participants felt that people “felt sorry” for them or were “ignorant” or “nosey,” and some felt that people were overly helpful, to a fault [87]. |
| 3a) Enacting management strategies: Institutional admissions | Undergoing acute care | The criticism expressed by the men concerned, for example, the long waiting hours at the emergency department before being admitted to the ward and also the way in which treatment was effectuated [62]. |
|  | Undergoing inpatient rehabilitation | His stay on the rehabilitation unit was of approximately 3 weeks duration, in which time he received physiotherapy for mobility and arm function, and occupational therapy to improve his ability to dress himself. By the time he was discharged, Robert could transfer between a chair and bed independently, and could mobilize with the help of one person and a walking frame [77]. |
|  | Fitting into ward routines | During the few days the participants were in the ward, there were a lot of new things to consider. Apart from facing the fact that they had had a stroke, they had to become aware of the daily routines in the ward and try to understand and follow them. This concerned, for example, hours when meals were served, what time the doctors’ rounds took place, what nurse and nurse assistant were responsible for their specific care, how and when it was appropriate to call for help, when the therapists were present at the ward and the staff’s expectations of their rehabilitation and outcome [62]. |
|  | Loss of autonomy and dignity as an inpatient | Loss of control over personal care (toileting, showering, eating) was described as particularly confronting. Almost all participants described being emotionally upset at the ‘loss of dignity’ they encountered during hospitalization [93]. |
|  | Unfamiliar or unpleasant surroundings on the ward | The participants spoke of the overwhelming boredom they experienced in the rehabilitation setting [72]. |
|  | Admission to a care home | In comparison with hospital, patients experience a personal atmosphere and receive more physiotherapy, occupational therapy and speech therapy which also facilitate autonomy [111]. |
|  | Learning self care skills to prepare for discharge | Patients take stock of the situation and indicate their priorities in learning. Learning self-care skills requires their own effort [111]. |
| 3b) Enacting management strategies: Managing stroke in the community | Discharge from hospital | The initial period after discharge was, for some of the stroke survivors, filled with practical problems because they did not feel adequately prepared to handle their daily lives and unexpected bodily reactions. As patients they had handed over the responsibility for the care of their stroke and related problems to the health care professionals. After being discharged from hospital to their homes they were left to manage by themselves in their former social context [52]. |
|  | Poor access to services in the community | Approximately half of the participants had, at some stage, received support from a psychologist, psychiatrist or counsellor in coping with their stroke, but this support was described as difficult to access and not available when most needed [93]. |
|  | Undergoing rehabilitation programmes in the community | He carried on with the work rehabilitation he had been assigned although he found it exhausting and totally meaningless. He saw no other solution [52]. |
|  | Taking and managing risks during rehabilitation | One aspect of rehabbing self that was reported by several participants was taking risks. This was a strategy employed in the phase of rehabbing self. It was identified as trying something without a guarantee that it was possible or would work [92]. |
|  | Reaching goals | Participants described that the process of community reintegration up to one year post stroke, rehabilitation involved transitioning between a series of goals: gaining physical function, establishing independence and adjusting expectations to get back to real living [82]. |
|  | Establishing and adhering to a medication regime | Almost all participants in the high adherence group recognized the importance of having a stable routine for taking their medication, and most had found a system that worked well for them. Taking their tablets was often spontaneously described as ‘routine’, ‘natural’ or a ‘habit’ and was consistently cued to particular daily activities such as showering or eating meals [7]. |
|  | Enduring medication side effects | Some low adherers also reported bad side effects of their medication. However, unlike high adherers experiencing side effects, who tended to persist with their medication or sought an alternative medicine from their general practitioner, patients in the low adherence group were more likely to alter their dosage or stop taking their tablets altogether to counteract any negative reactions [7]. |
|  | Managing risk factors | A few appeared to have been proactive about controlling their risk and talked about getting information or taking action to control blood pressure. A couple reported having faith in their treatment [73]. |
|  | Adjusting diet | The diets of participants varied considerably. Since the stroke, three survivors had adopted cholesterol-lowering diets, one had excluded wheat products and another had espoused a ‘healthy’ diet (reduced fat, increased fruit and vegetable intake). One now drank less alcohol and had lost weight, another drank more  and had gained three stone. Two diabetics described how  they now used sugar to ‘keep your strength up’ [71]. |
|  | Managing eating difficulties | Preventing things happening could mean to take small bites instead of big ones, to avoid that food getting stuck when too much food was put into the mouth at one time, and discovering which type of food they could choose [67]. |
|  | Managing psychological difficulties | The use of relaxation exercises and tapes provided relief from emotional distress, particularly in the hospital environment [93]. |
|  | Managing pain | Satisfaction was described as a result of various treatments offered by the professionals, such as aquatic exercise and massage by Transcutaneous Electrical Nerve Stimulation (TENS), also as a result of receiving help in changing their body position at night [57]. |
|  | Regaining communication skills | Participants with communication difficulties described these as overwhelming and many became preoccupied with regaining their communication abilities, to the extent that other needs or physical symptoms were ignored [93]. |
|  | Taking physical exercise | Physical exercise was reported by participants with nociceptive pain in order to prevent pain by stretching their shoulder/arm using training equipment, or aquatic exercise. In order to relieve their pain, participants with central pain did perform physical stretching exercises, but were careful to avoid overexertion. The participants with tension-type headache reported that they went out for a walk to get some fresh air in an attempt to relieve the headache [56]. |
|  | Managing co-morbidities | Extra coping strategies, such as pharmaceutical use and activity avoidance, were used by participants to deal with the additional difficulties associated with having arthritis while recovering from a stroke, in order to remain engaged [86]. |
|  | Making adaptations to the home environment or finding new accommodation | Another problem that PEG feeding could pose was the space occupied by supplies and equipment; this hampered mobility of a wheelchair dependent stroke survivor in a tiny bungalow [71]. |
|  | Enduring inadequate home services | The self –care activities that were not possible for many of the stroke survivors included taking a bath or shower, brushing teeth and having sexual intercourse. This caused frustration and humiliation for them. One described not being able to have enough assistance with taking a shower because of the regulations of the attendant care service [90]. |
|  | Coping with multiple health related appointments | Returning home was overwhelming. Participants were confused, busy and tired because of health-related appointments and visits from friends and family [82]. |
| 3c) Enacting management strategies: Reintegrating into society | Returning to driving or negotiating new methods of transport | For those that did resume driving during the first year post rehabilitation, it was critical to regaining control and independence [82]. |
|  | Returning to work | The occupational therapist was involved in this RTW process and arranged meetings with the workplace, the manager, and in one case also with co-workers. However, only two of the six informants had experienced such a liaison. It was more common that no preparation at all had taken place for return to work when they were discharged from the hospital [54]. |
|  | Acquiring mobility and technical aids | Despite the fear of becoming dependent, however, some participants had been able to change habitual ways with the use of technical aids. Aids for increasing mobility, such as walking canes, walkers, and wheelchairs seemed to  have a degree of acceptability and in regard to clothing, participants had also begun to introduce certain adaptations [68]. |
|  | Negotiating environmental barriers to wheelchair use | The wheelchair helped them with distances when they were tired, but was seen as a nuisance by others because of space restrictions, getting the wheelchair through narrow doorways in the home and community was a problem [90]. |
|  | Managing financial difficulties | The stroke survivors in this study did not have the economic resources to modify their existing living environment, except for a few renovations to their homes. The majority relied on low technology devices to manage in their homes [90]. |
|  | Negotiating government benefit systems | Organizations were frequently viewed as threatening or oppositional, setting up road blocks to needed resources. One of the primary bureaucracies that survivors contended with relates to obtaining disability payments, often an arduous task [107]. |
| 3d) Enacting management strategies: Adjusting to life after stroke | New daily structure to accommodate illness management | Initially trying pre-stroke behaviours for occupational performance, the participants in this study soon learned to try modifications of their usual routines as an adaptive response to the challenge of fatigue in their daily roles. The context of their lives had to be reconfigured to accommodate this powerful phenomenon of fatigue [106]. |
|  | Relearning ways of doing familiar tasks | Over time, some participants were able to adapt to a new way of life. This entailed learning how to do new things, relearning abilities (such as spelling, communication, walking) and continued commitment to the rehabilitation process [93]. |
|  | Planning activities ahead of time | The study data suggest that problems with performance of habits after stroke, previously used to accomplish daily occupations had been disrupted. The participants had to plan ahead, think, and reflect when carrying out a daily occupation simply to manage their everyday lives. Despite living in a familiar home environment, many of the daily occupations that participants had previously done no longer seemed to be easy to perform and even if repeated often they didn’t became habitual. Participants felt that, no matter what they did, they had to think about and plan their daily occupations in advance [68]. |
|  | Adopting strategies to deal with physical disabilities | Practical solutions to physical limitations, such as carrying a communication card, making lists to assist memory or finding new ways of dressing independently were empowering [93]. |
|  | Adopting strategies to deal with cognitive disabilities | In order to try to adapt to her memory problems following her stroke, Mrs. N.M. developed an extensive filing system of her various interests, which catalogues the various things she needs to know about different tasks and activities. However, her husband is very critical of this system and tries to remove its presence from the house [83]. |
|  | Searching for a sense of self | More often than not, individuals struggle to participate in their daily lives, in an attempt to reach their pre-stroke level of participation [106]. |
|  | Developing acceptance | One prominent theme among those women who had apparently lost hope was acceptance. Although the loss of hope is often associated with depression, these women described an acceptance of their limitations [89]. |
|  | Enduring a plateau in recovery | During rehabilitation some patients experience a status quo (levelling out in their rate or degree of progress) regarding the dimension self-care [111]. |
|  | Changing expectations and examining priorities over the recovery period | Gradually, they learned that their initial timeframe was too optimistic. The `horizon of time’ shifted from a few days to a few weeks, to half a year, to within a year, and so forth. This change occurred as the patients gained experience with their bodies and the slowness of improvement [108]. |
| 4) Reflecting on management | Decision making about treatments | Discontinuing medication, both prescribed and non-prescription analgesics, was reported by participants in all groups because of insufficient pain relief and side effects or fear of side effects [56]. |
|  | Shared decision making about treatments | Although decision-making about their own treatment is difficult for most patients, they valued shared decision-making, e.g. about destination and moment of discharge, recreation, meals, therapy schedules, care routines, and aids [111]. |
|  | Monitoring progress in recovery | Mr Neville an 80 year old man set himself the target of walking unaided by the time he left hospital….He kept a diary of is progress which he made available to the research team…Stroke survivors presented their goals as an aspiration or a challenge. By definitions they were activities that were important or valued and they could not perform. The goals were often related to the recovery of specific skills that were part of personal competence and identity [75]. |
|  | Gauging recovery by comparing self to others | Comparisons with other patients - one patient took the example of a stroke patient who recovered well as a source of confidence in her own recovery. Other patients, however, reported feeling depressed at the fact they were not rehabilitating as well as other patients [76]. |
|  | Self monitoring for further signs of stroke | Several survivors reported that they were more sensitive to their body and were vigilant in seeking to identify any body signs or changes that could be an early warning of another stroke. Mrs Dean and Mrs Garrett were both sensitive to changes close to their brain, but they reacted in different ways to episodes of facial numbness [75]. |
|  | Maintaining confidence in care plan | Whenever participants were transitioning to a new goal, for example from gaining physical function while in rehabilitation to establishing independence within the community, the experience was marked by a decrease in confidence. This decreased confidence was the result of being faced with challenges not experienced since their stroke [82]. |
|  | Keeping up to date with new treatments | One man told a story about when he had asked the doctor for more protective treatment against new strokes [62]. |
