# Supplementary material for: Uncovering Treatment Burden as a Key Concept for Stroke Care: A Systematic Review of Qualitative Research
Source: PLoS Med. 2013 Jun 25;10(6):e1001473. doi: 10.1371/journal.pmed.1001473 (PMC3692487; doi:10.1371/journal.pmed.1001473)
Supplement: Text S1 — Search strategy. Details of the strategies employed for searching Medline, Embase, PsycINFO, CINAHL, and Scopus databases. (DOC) [file pmed.1001473.s007.doc]

Text S1 - Search strategy.

*Details of the strategies employed for searching Medline, Embase, PsycINFO, CINAHL & Scopus databases.*

**Ovid MEDLINE(R) In-Process & Other Non-Indexed Citations and Ovid MEDLINE(R) 1948 to Present # Searches Results Search Type**

1 *cerebrovascular disorders/ or exp *brain ischemia/ or exp "*intracranial embolism and thrombosis"/ or exp *intracranial hemorrhages/ or exp *stroke/

2 *stroke/co, di, dh, dt, nu, pc, px, rh, th

3 (cerebrovascular disorders/co, di, dh, dt, nu, pc, px, rh, th or exp brain ischemia/co, di, dh, dt, nu, pc, px, rh, th or exp "intracranial embolism and thrombosis"/co, di, dh, dt, nu, pc, px, rh, th or exp intracranial hemorrhages/co, di, dh, dt, nu, pc, px, rh, th) and stroke*.mp. [mp=protocol supplementary concept, rare disease supplementary concept, title, original title, abstract, name of substance word, subject heading word, unique identifier]

4 1 or 2 or 3

5 limit 4 to (english language and humans and yr="2000 - 2013")

6 (burden* adj2 (treat* or therap*)).mp. [mp=protocol supplementary concept, rare disease supplementary concept, title, original title, abstract, name of substance word, subject heading word, unique identifier]

7 activities of daily living/ or health behavior/ or health, knowledge, attitudes, practice/ or lifestyle/ or occupational therapy/ or palliat*.mp. or patient education as topic/ or exp rehabilitation/ or exp physical therapy modalities/ or self care/ or barthel*.mp. [mp=protocol supplementary concept, rare disease supplementary concept, title, original title, abstract, name of substance word, subject heading word, unique identifier]

8 Disabled Persons/ or Disability Evaluation/ or work capacity evaluation/ or functional disability.mp.

9 social support/ or exp family/px or exercise/ or "physical education and training"/ or physical endurance/ or fatigue/ or physical fitness/ or long term care/

10 exp Gait/ or exp Gait Disorders, Neurologic/ or exp Accidental Falls/

11 self administration/ or (treatment adj3 (regimen* or regime* or tasks or work)).mp. or unnecessary*.mp. or unmet.mp. [mp=protocol supplementary concept, rare disease supplementary concept, title, original title, abstract, name of substance word, subject heading word, unique identifier

12 exp cognition disorders/ or independen*.mp. or dependenc*.mp. or socioeconomic factors/ or life change events/ [mp=protocol supplementary concept, rare disease supplementary concept, title, original title, abstract, name of substance word, subject heading word, unique identifier]

13 5 and 6

14 5 and 7

15 5 and 8

16 5 and 9

17 5 and 10

18 5 and 11

19 5 and 12

20 5 and (travel*.mp. or automobile driving/ or mobility limitation/ or transportation/) [mp=protocol supplementary concept, rare disease supplementary concept, title, original title, abstract, name of substance word, subject heading word, unique identifier]

21 5 and (recover* or challeng* or goal*).mp. [mp=protocol supplementary concept, rare disease supplementary concept, title, original title, abstract, name of substance word, subject heading word, unique identifier]

22 5 and (interpersonal relations/ or social isolation/ or cost of illness/ or schedul*.mp.) [mp=protocol supplementary concept, rare disease supplementary concept, title, original title, abstract, name of substance word, subject heading word, unique identifier]

23 or/13-22

24 (focus group* or ethnograph* or phenomenol* or observation* or (grounded adj theory) or (framework adj analysis) or (thematic adj analysis) or (constant adj comparison)).mp. [mp=protocol supplementary concept, rare disease supplementary concept, title, original title, abstract, name of substance word, subject heading word, unique identifier]

25 adaptation, psychological/ or adheren*.mp. or nonadheren*.mp. or patient compliance/ or noncomplian*.mp. or inconvenien*.mp. or negotiat*.mp. or (patient adj2 (care or experience or understand* or expectation* or perspective*)).mp. or patient satisfaction/ or personal autonomy/ or physician-patient relations/ or professional-patient relations/ or dissatis*.mp. or quality of life/ [mp=protocol supplementary concept, rare disease supplementary concept, title, original title, abstract, name of substance word, subject heading word, unique identifier]

26 self concept/ or self care/ or self-management/ or suffer*.mp. [mp=protocol supplementary concept, rare disease supplementary concept, title, original title, abstract, name of substance word, subject heading word, unique identifier]

27 attitude/ or attitude to health/

28 (questionnaire* or survey* or qualitative* or interview*).mp. [mp=protocol supplementary concept, rare disease supplementary concept, title, original title, abstract, name of substance word, subject heading word, unique identifier]

29 23 and (25 or 26 or 27) and (24 or 28)

30 23 and (exp leisure activities/ or health status/ or well-being.mp.) and (24 or 28) [mp=protocol supplementary concept, rare disease supplementary concept, title, original title, abstract, name of substance word, subject heading word, unique identifier]

31 29 or 30

32 Health Services Accessibility/

33 23 and (32 or continuity of patient care/ or disrupt*.mp.) and (24 or 28) [mp=protocol supplementary concept, rare disease supplementary concept, title, original title, abstract, name of substance word, subject heading word, unique identifier]

34 5 and (communication* or literac*).mp. and (24 or 28) [mp=protocol supplementary concept, rare disease supplementary concept, title, original title, abstract, name of substance word, subject heading word, unique identifier]

35 31 or 33 or 34

36 5 and sickness impact profile/

37 5 and needs assessment/

38 35 or 36 or 37

**EMBASE 1988 to 2013 Week 08 # Searches Results Search Type**

1 *cerebrovascular disorders/ or exp *brain ischemia/ or exp "*intracranial embolism and thrombosis"/ or exp *intracranial hemorrhages/ or exp *stroke/

2 *stroke/co, di, dm, dt, rh, th

3 cerebrovascular disorders/co, di, dm, dt, rh, th or brain ischemia/co, di, dm, dt, rh, th or exp "intracranial embolism and thrombosis"/co, di, dm, dt, dm, rh, th or exp intracranial hemorrhages/co, di, dm, dt, rh, th

4 1 or 2 or 3

5 limit 4 to (english language and humans and yr="2000 - 2013")

6 ((burden* adj2 (treat* or therap*)) or hassle* or medication adherence or medication compliance or medication concordance or physiotherapy or ocupational therapy).mp. or rehabilitation/ [mp=title, abstract, subject headings, heading word, drug trade name, original title, device manufacturer, drug manufacturer]

7 activities of daily living/ or health behavior/ or health, knowledge, attitudes, practice/ or lifestyle/ or occupational therapy/ or palliat*.mp. or patient education as topic/ or exp rehabilitation/ or exp physical therapy modalities/ or self care/ or barthel*.mp. [mp=title, abstract, subject headings, heading word, drug trade name, original title, device manufacturer, drug manufacturer]

8 Disabled Persons/ or Disability Evaluation/ or work capacity evaluation/ or functional disability.mp.

9 social support/ or exp family/px or exercise/ or "physical education and training"/ or physical endurance/ or fatigue/ or physical fitness/ or long term care/

10 exp Gait/ or exp Gait Disorders, Neurologic/ or exp Accidental Falls/

11 self administration/ or (treatment adj3 (regimen* or regime* or tasks or work)).mp. or unnecessary*.mp. or unmet.mp. [mp=title, abstract, subject headings, heading word, drug trade name, original title, device manufacturer, drug manufacturer]

12 exp cognition disorders/ or independen*.mp. or dependenc*.mp. or socioeconomic factors/ or life change events/ [mp=title, abstract, subject headings, heading word, drug trade name, original title, device manufacturer, drug manufacturer]

13 5 and 6

14 5 and 7

15 5 and 8

16 5 and 9

17 5 and 10

18 5 and 11

19 5 and 12

20 5 and (travel*.mp. or automobile driving/ or mobility limitation/ or transportation/) [mp=title, abstract, subject headings, heading word, drug trade name, original title, device manufacturer, drug manufacturer]

21 5 and (recover* or challeng* or goal*).mp. [mp=title, abstract, subject headings, heading word, drug trade name, original title, device manufacturer, drug manufacturer]

22 5 and (interpersonal relations/ or social isolation/ or cost of illness/ or schedul*.mp.) [mp=title, abstract, subject headings, heading word, drug trade name, original title, device manufacturer, drug manufacturer]

23 or/13-22

24 (focus group* or ethnograph* or phenomenol* or observation* or (grounded adj theory) or (framework adj analysis) or (thematic adj analysis) or (constant adj comparison)).mp. [mp=title, abstract, subject headings, heading word, drug trade name, original title, device manufacturer, drug manufacturer]

25 adaptation, psychological/ or adheren*.mp. or nonadheren*.mp. or patient compliance/ or noncomplian*.mp. or inconvenien*.mp. or negotiat*.mp. or (patient adj2 (care or experience or understand* or expectation* or perspective*)).mp. or patient satisfaction/ or personal autonomy/ or physician-patient relations/ or professional-patient relations/ or dissatis*.mp. or quality of life/ [mp=title, abstract, subject headings, heading word, drug trade name, original title, device manufacturer, drug manufacturer]

26 self concept/ or self care/ or self-management/ or suffer*.mp. [mp=title, abstract, subject headings, heading word, drug trade name, original title, device manufacturer, drug manufacturer]

27 attitude/ or attitude to health/

28 (questionnaire* or survey* or qualitative* or interview*).mp. [mp=title, abstract, subject headings, heading word, drug trade name, original title, device manufacturer, drug manufacturer]

29 23 and (25 or 26 or 27) and (24 or 28)

30 23 and (exp leisure activities/ or health status/ or well-being.mp.) and (24 or 28) [mp=title, abstract, subject headings, heading word, drug trade name, original title, device manufacturer, drug manufacturer]

31 29 or 30

32 Health Services Accessibility/

33 23 and (32 or continuity of patient care/ or disrupt*.mp.) and (24 or 28) [mp=title, abstract, subject headings, heading word, drug trade name, original title, device manufacturer, drug manufacturer]

34 5 and (communication* or literac*).mp. and (24 or 28) [mp=title, abstract, subject headings, heading word, drug trade name, original title, device manufacturer, drug manufacturer]

35 31 or 33 or 34

36 5 and sickness impact profile/

37 5 and needs assessment/

38 35 or 36 or 37

39 limit 38 to (human and yr="2000 - 2013")

40 39 not case report/

41 ((experience or recovery or service or patient*) adj3 stroke*).mp. [mp=title, abstract, subject headings, heading word, drug trade name, original title, device manufacturer, drug manufacturer]

42 (exercise or lifestyle or patient education* or social support or social isolation or self care or burden or community support or cost of illness or drug costs or imipact or inconvenience or negotiat* or patient experience or patient perspective or patient preference or patient satisfaction or dissatis*).mp. or professional-patient relations/ or physician patient relations.mp. or quality of life.mp. or recovery*.mp. or suffer*.mp. [mp=title, abstract, subject headings, heading word, drug trade name, original title, device manufacturer, drug manufacturer]

43 5 and (41 or 42)

44 43 and 24

45 39 or 44

46 (*cerebrovascular disorders/ or exp *brain ischemia/ or exp *"*intracranial embolism and thrombosis"/ or exp *intracranial hemorrhages/ or exp *stroke/) and 45

**PsycINFO 1987 to Feb Week 3 2013 # Searches Results Search Type**

1 *cerebrovascular disorders/ or exp *brain ischemia/ or exp "*intracranial embolism and thrombosis"/ or exp *intracranial hemorrhages/ or exp *stroke/

2 [*stroke/co, di, dm, dt, rh, th]

3 [cerebrovascular disorders/co, di, dm, dt, rh, th or brain ischemia/co, di, dm, dt, rh, th or exp "intracranial embolism and thrombosis"/co, di, dm, dt, dm, rh, th or exp intracranial hemorrhages/co, di, dm, dt, rh, th]

4 1 or 2 or 3

5 limit 4 to (english language and humans and yr="2000 - 2013") [Limit not valid in PsycINFO; records were retained]

6 ((burden* adj2 (treat* or therap*)) or hassle* or medication adherence or medication compliance or medication concordance or physiotherapy or ocupational therapy).mp. or rehabilitation/ [mp=title, abstract, heading word, table of contents, key concepts]

7 activities of daily living/ or health behavior/ or health, knowledge, attitudes, practice/ or lifestyle/ or occupational therapy/ or palliat*.mp. or patient education as topic/ or exp rehabilitation/ or exp physical therapy modalities/ or self care/ or barthel*.mp. [mp=title, abstract, heading word, table of contents, key concepts]

8 Disabled Persons/ or Disability Evaluation/ or work capacity evaluation/ or functional disability.mp.

9 social support/ or exp family/px or exercise/ or "physical education and training"/ or physical endurance/ or fatigue/ or physical fitness/ or long term care/

10 exp Gait/ or exp Gait Disorders, Neurologic/ or exp Accidental Falls/

11 self administration/ or (treatment adj3 (regimen* or regime* or tasks or work)).mp. or unnecessary*.mp. or unmet.mp. [mp=title, abstract, heading word, table of contents, key concepts]

12 exp cognition disorders/ or independen*.mp. or dependenc*.mp. or socioeconomic factors/ or life change events/ [mp=title, abstract, heading word, table of contents, key concepts]

13 5 and 6

14 5 and 7

15 5 and 8

16 5 and 9

17 5 and 10

18 5 and 11

19 5 and 12

20 5 and (travel*.mp. or automobile driving/ or mobility limitation/ or transportation/) [mp=title, abstract, heading word, table of contents, key concepts]

21 5 and (recover* or challeng* or goal*).mp. [mp=title, abstract, heading word, table of contents, key concepts]

22 5 and (interpersonal relations/ or social isolation/ or cost of illness/ or schedul*.mp.) [mp=title, abstract, heading word, table of contents, key concepts]

23 or/13-22

24 (focus group* or ethnograph* or phenomenol* or observation* or (grounded adj theory) or (framework adj analysis) or (thematic adj analysis) or (constant adj comparison)).mp. [mp=title, abstract, heading word, table of contents, key concepts]

25 adaptation, psychological/ or adheren*.mp. or nonadheren*.mp. or patient compliance/ or noncomplian*.mp. or inconvenien*.mp. or negotiat*.mp. or (patient adj2 (care or experience or understand* or expectation* or perspective*)).mp. or patient satisfaction/ or personal autonomy/ or physician-patient relations/ or professional-patient relations/ or dissatis*.mp. or quality of life/ [mp=title, abstract, heading word, table of contents, key concepts]

26 self concept/ or self care/ or self-management/ or suffer*.mp. [mp=title, abstract, heading word, table of contents, key concepts]

27 attitude/ or attitude to health/

28 (questionnaire* or survey* or qualitative* or interview*).mp. [mp=title, abstract, heading word, table of contents, key concepts]

29 23 and (25 or 26 or 27) and (24 or 28)

30 23 and (exp leisure activities/ or health status/ or well-being.mp.) and (24 or 28) [mp=title, abstract, heading word, table of contents, key concepts]

31 29 or 30

32 Health Services Accessibility/

33 23 and (32 or continuity of patient care/ or disrupt*.mp.) and (24 or 28) [mp=title, abstract, heading word, table of contents, key concepts]

34 5 and (communication* or literac*).mp. and (24 or 28) [mp=title, abstract, heading word, table of contents, key concepts]

35 31 or 33 or 34

36 5 and sickness impact profile/

37 5 and needs assessment/

38 35 or 36 or 37

39 limit 38 to (human and yr="2000 - 2013")

40 39 not case report/

41 ((experience or recovery or service or patient*) adj3 stroke*).mp. [mp=title, abstract, heading word, table of contents, key concepts]

42 (exercise or lifestyle or patient education* or social support or social isolation or self care or burden or community support or cost of illness or drug costs or imipact or inconvenience or negotiat* or patient experience or patient perspective or patient preference or patient satisfaction or dissatis*).mp. or professional-patient relations/ or physician patient relations.mp. or quality of life.mp. or recovery*.mp. or suffer*.mp. [mp=title, abstract, heading word, table of contents, key concepts]

43 5 and (41 or 42)

44 43 and 24

45 39 or 44

46 limit 45 to all journals

**CINAHL**

S22 or S27 Limiters - Published Date from: 20000101-20131231; English Language; Human

Search modes - Boolean/Phrase

View Results View Details Edit Interface - EBSCOhost

Search Screen - Advanced Search

Database - CINAHL

S28 S22 or S27 Search modes - Boolean/Phrase

View Results View Details Edit Interface - EBSCOhost

Search Screen - Advanced Search

Database - CINAHL

S27 S1 and S26 and S11 Search modes - Boolean/Phrase

View Results View Details Edit Interface - EBSCOhost

Search Screen - Advanced Search

Database - CINAHL

S26 S23 or S24 or S25 Search modes - Boolean/Phrase

View Results View Details Edit Interface - EBSCOhost

Search Screen - Advanced Search

Database - CINAHL

S25 disappoint* or dissatisf* or emotional or eating or frustrat* Search modes - Boolean/Phrase

View Results View Details Edit Interface - EBSCOhost

Search Screen - Advanced Search

Database - CINAHL

S24 (MH "Quality of Life+") OR (MH "Quality of Working Life") OR (MH "Quality of Life (Iowa NOC)") OR (MH "Attitude to Life") Search modes - Boolean/Phrase

View Results View Details Edit Interface - EBSCOhost

Search Screen - Advanced Search

Database - CINAHL

S23 function* or negotiat* or patient* Search modes - Boolean/Phrase

View Results View Details Edit Interface - EBSCOhost

Search Screen - Advanced Search

Database - CINAHL

S22 S13 or S21 Search modes - Boolean/Phrase

View Results View Details Edit Interface - EBSCOhost

Search Screen - Advanced Search

Database - CINAHL

S21 S1 and S11 and S20 Search modes - Boolean/Phrase

View Results View Details Edit Interface - EBSCOhost

Search Screen - Advanced Search

Database - CINAHL

S20 S14 or S15 or S16 or S18 or S19 Search modes - Boolean/Phrase

View Results View Details Edit Interface - EBSCOhost

Search Screen - Advanced Search

Database - CINAHL

S19 (MH "Recovery") Search modes - Boolean/Phrase

View Results View Details Edit Interface - EBSCOhost

Search Screen - Advanced Search

Database - CINAHL

S18 (MH "Patient Satisfaction") Search modes - Boolean/Phrase

View Results View Details Edit Interface - EBSCOhost

Search Screen - Advanced Search

Database - CINAHL

S17 "patient experience" or "patient satisfaction" or "patient dissatisf* Search modes - Boolean/Phrase

View Results View Details Edit Interface - EBSCOhost

Search Screen - Advanced Search

Database - CINAHL

S16 (MH "Patient Education+") OR (MH "Patient Discharge Education") OR (MH "Patient Education (Iowa NIC) (Non-Cinahl)+") Limiters - Published Date from: 20000101-20131231; English Language

Search modes - Boolean/Phrase

View Results View Details Edit Interface - EBSCOhost

Search Screen - Advanced Search

Database - CINAHL

S15 (MH "Patient Care Plans+") OR (MH "Discharge Planning+") Limiters - Published Date from: 20000101-20131231; English Language

Search modes - Boolean/Phrase

View Results View Details Edit Interface - EBSCOhost

Search Screen - Advanced Search

Database - CINAHL

S14 (MH "Life Style+") Limiters - Published Date from: 20000101-20131231; English Language

Search modes - Boolean/Phrase

View Results View Details Edit Interface - EBSCOhost

Search Screen - Advanced Search

Database - CINAHL

S13 S9 and S11 Limiters - Published Date from: 20000101-20131231; English Language

Search modes - Boolean/Phrase

View Results View Details Edit Interface - EBSCOhost

Search Screen - Advanced Search

Database - CINAHL

S12 S9 and S11 Search modes - Boolean/Phrase

View Results View Details Edit Interface - EBSCOhost

Search Screen - Advanced Search

Database - CINAHL

S11 (MH "Qualitative Studies+") OR (MH "Clinical Nursing Research") OR (MH "Clinical Research+") Search modes - Boolean/Phrase

View Results View Details Edit Interface - EBSCOhost

Search Screen - Advanced Search

Database - CINAHL

S10 ch Search modes - Boolean/Phrase

View Results (0) View Details Edit Interface - EBSCOhost

Search Screen - Advanced Search

Database - CINAHL

S9 S1 and S8 Search modes - Boolean/Phrase

View Results View Details Edit Interface - EBSCOhost

Search Screen - Advanced Search

Database - CINAHL

S8 S2 or S3 or S4 or S5 or S6 or S7 Search modes - Boolean/Phrase

View Results View Details Edit Interface - EBSCOhost

Search Screen - Advanced Search

Database - CINAHL

S7 (MH "Social Support (Iowa NOC)") OR (MH "Norbeck Social Support Questionnaire") OR (MH "Social Support Index") OR (MH "Support, Psychosocial+") OR (MH "Coping Support (Saba CCC)") Search modes - Boolean/Phrase

View Results View Details Edit Interface - EBSCOhost

Search Screen - Advanced Search

Database - CINAHL

S6 lifestyle Search modes - Boolean/Phrase

View Results View Details Edit Interface - EBSCOhost

Search Screen - Advanced Search

Database - CINAHL

S5 (MH "Health Knowledge") Search modes - Boolean/Phrase

View Results View Details Edit Interface - EBSCOhost

Search Screen - Advanced Search

Database - CINAHL

S4 (MH "Health Behavior+") OR (MH "Health Behavior Component (Saba CCC)+") OR (MH "Health Seeking Behavior Alteration (Saba CCC)") OR (MH "Domain IV: Health-Related Behaviors Domain (Omaha)+") OR (MH "Health Behavior (Iowa NOC) (Non-Cinahl)+") OR (MH "Health Knowledge and Behavior (Iowa NOC) (Non-Cinahl)+") OR (MH "Health Promoting Behavior (Iowa NOC)") Search modes - Boolean/Phrase

View Results View Details Edit Interface - EBSCOhost

Search Screen - Advanced Search

Database - CINAHL

S3 "burden of treatment" or "burden of therapy" or hassle* or inconvenien* or "treatment burden" Search modes - Boolean/Phrase

View Results View Details Edit Interface - EBSCOhost

Search Screen - Advanced Search

Database - CINAHL

S2 (MH "Altered Activities of Daily Living (NANDA) (Non-Cinahl)+") OR (MH "Self Care: Activities of Daily Living (Iowa NOC)") OR (MH "Self-Care: Instrumental Activities of Daily Living (Iowa NOC)") OR (MH "Activities of Daily Living+") OR (MH "Activities of Daily Living (Saba CCC)") OR (MH "Activities of Daily Living Alteration (Saba CCC)") OR (MH "Instrumental Activities of Daily Living (Saba CCC)") OR (MH "Instrumental Activities of Daily Living Alteration (Saba CCC)") Search modes - Boolean/Phrase

View Results View Details Edit Interface - EBSCOhost

Search Screen - Advanced Search

Database - CINAHL

S1 (MM "Stroke") OR (MM "Stroke Patients") Search modes - Boolean/Phrase

View Results View Details Edit

 Search ID#  Search Terms  Search Options  Actions 
   S15   S9 or S14   Search modes - Boolean/Phrase
   View Results 
   S14   S1 and S11   Limiters - Published Date from: 20000101-20131231; English Language; Peer Reviewed; Research Article

   View Results 
   S13   S1 and S11   Limiters - Published Date from: 20000101-20131231; English Language; Peer Reviewed; Research Article

   S12   S1 and S11   Search modes - Boolean/Phrase
   View Results
   S11   (MH "patient centered care") or (MH "community health services") or (MH "health resource utilization") or (MH "community reintegration") or (MH "patient attitudes") or (MH "recovery") or (MH "patient satisfaction")   Search modes - Boolean/Phrase

   View Results
   S10   S1 and S6   Search modes - Boolean/Phrase
   View Results 
   S9   S6 and S7   Limiters - Published Date from: 20000101-20131231; English Language; Peer Reviewed; Exclude MEDLINE records

   View Results 
   S8   S6 and S7   Search modes - Boolean/Phrase
   View Results 
   S7   S1 or S2   Search modes - Boolean/Phrase
   View Results 
   S6   S3 or S4 or S5   Search modes - Boolean/Phrase
   View Results 
   S5   (MH "Interviews+")   Search modes - Boolean/Phrase
   View Results 
   S4   (MH "Scales") OR (MH "Questionnaires+")   Search modes - Boolean/Phrase
   S3   (MH "Qualitative Studies+")   Search modes - Boolean/Phrase
   View Results 
   S2   (MM "Stroke")   Search modes - Boolean/Phrase
   View Results 
   S1   (MH "Stroke Patients")   Search modes - Boolean/Phrase

**SCOPUS**TITLE-ABS-KEY-AUTH(stroke AND burden* AND (rehab* OR poststroke)) AND PUBYEAR AFT 1999
TITLE-ABS-KEY((stroke* OR poststroke OR "cerebrovascular accident*")) AND ((rehabilitat* OR satisfact* OR dissatisfact* OR recover* OR surviv* OR work OR occupation* OR "return to work" OR community OR support OR recover* OR independen* OR dependen* OR driving OR coping OR frustrat* OR attitude* OR communicat* OR activities* OR adapt* OR burden* OR self OR impact OR inconven* OR mood* OR emotion* OR sexual* OR eating OR dysphag* OR dysfunction* OR disappoint* OR expectation* OR intimacy OR incontinen* OR depress* OR fatigue* OR preference OR quality OR suffer* OR unmet) AND (qualitative* OR survey* OR interview* OR questionnaire* OR perspective* OR scale* OR narrative* OR focus OR observational OR framework OR thematic OR "grounded theory" OR population* OR cohort* OR follow*)) AND TITLE(aphasi* OR "post-stroke" OR poststroke OR (stroke AND (surviv* OR recover* OR rehabil* OR needs OR goal* OR patient* OR surviv*))) AND PUBYEAR AFT 1999 AND LANGUAGE(english) AND NOT (PMID(1* OR 2* OR 3* OR 4* OR 5* OR 6* OR 7* OR 8* OR 9*)) AND NOT TITLE-ABS-KEY("case report*" OR mice OR rats) AND (EXCLUDE(DOCTYPE, "ip")) AND (EXCLUDE(SUBJAREA, "ENGI"))
